# Anti-Biofilm and Antivirulence Activities of Metabolites from *Plectosphaerella cucumerina* against *Pseudomonas aeruginosa*

**DOI:** 10.3389/fmicb.2017.00769

**Published:** 2017-05-03

**Authors:** Jinwei Zhou, Shiyuan Bi, Hongjuan Chen, Tongtong Chen, Rui Yang, Minghui Li, Yonghong Fu, Ai-Qun Jia

**Affiliations:** ^1^School of Environmental and Biological Engineering, Nanjing University of Science and TechnologyNanjing, China; ^2^State Key Laboratory of Pharmaceutical Biotechnology, Nanjing UniversityNanjing, China; ^3^State Key Laboratory of Marine Resource Utilization in South China Sea, Key Laboratory of Tropical Biological Resources of Ministry Education, Hainan UniversityHaikou, China

**Keywords:** *Plectosphaerella cucumerina*, phyllosphere fungus, *Pseudomonas aeruginosa*, biofilm, quorum sensing

## Abstract

This study reported the efficacy of the metabolites of *Plectosphaerella cucumerina*, one phyllosphere fungus from *Orychophragmus violaceus*, against *Pseudomonas aeruginosa* quorum sensing (QS) and QS-regulated biofilms. The minimum inhibitory concentration (MIC) of the ethyl acetate (EtOAc) extract from *P. cucumerina* against *P. aeruginosa* PAO1 was 1.25 mg mL^−1^. At sub-MIC concentrations, *P. cucumerina* extract (0.25–1 mg mL^−1^) not only inhibited biofilm formation but also disrupted preformed biofilms of *P. aeruginosa* PAO1 without affecting its growth. Fluorescence and scanning electron microscope (SEM) showed architectural disruption of the biofilms when treated with *P. cucumerina* metabolites. Further investigation demonstrated that metabolites in *P. cucumerina* attenuated the QS-dependent virulence factors. LC-MS/MS spectra coupled with experimentally standard samples suggested that patulin and emodin might act as the principal components possessing anti-biofilm and antivirulence activities. This is the first report of (1) the isolation of *P. cucumerina* from the phyllosphere of *O. violaceus* and (2) anti-biofilm, antivirulence, and biofilm disruption activities of this fungus. Thus, this study provides fascinating new pathways for screening antipathogenic agents.

## Introduction

Most pathogenic bacteria can form biofilms under diverse conditions (Hall-Stoodley and Stoodley, 2005). Among biofilm forming microbial pathogens, *Pseudomonas aeruginosa* is the most notorious bacterium causing chronic diseases in nature (Morita et al., 2014). The pathogenicity of *P. aeruginosa* is mainly owing to the biofilms, which make this organism more resistant to physical and chemical treatment (Imperi et al., 2014; Pompilio et al., 2015). Biofilm formation is a highly regulated process that is closely associated with quorum sensing (QS) (Qin et al., 2014). QS is a bacterial communication phenomenon that relies on secreted signals for coordinating group behaviors (Kim et al., 2015). N-acylated L-homoserine lactones (AHLs) are released as autoinducers to facilitate QS of Gram-negative bacteria (LaSarre and Federle, 2013). In many cases, QS signals were directly employed by bacteria to control biofilm formation and production of virulence factors, such as pyocyanin and rhamnolipids (Rutherford and Bassler, 2012). Pyocyanin induces oxidative stress and positively correlates with disease severity (Hunter et al., 2012). Rhamnolipids play vital roles in chronic infection by facilitating biofilm maturation and immune evasion (Zulianello et al., 2006).

*P. aeruginosa* owns three QS systems, *las, rhl*, and *pqs* (Kim et al., 2015). Each system possesses one transcriptional regulator and its related signals. The signals N-(3-oxododecanoyl)-L-homoserine lactone (3-oxo-C12-HSL, OdDHL) and N-butanoyl-L-homoserine lactone (C4-HSL, BHL) are produced by the *las* and *rhl* systems, and the autoinducer 2-heptyl-3-hydroxy-4(1*H*)-quinolone, known as *Pseudomonas* quinolone signal (PQS) from the *pqs* system (Chan et al., 2015; Liu et al., 2015). There is a regulatory hierarchy between these three systems, with the *rhl* and *pqs* systems regulated by the *las* system (Smith, 2003). The signals can bind their cognate transcriptional regulators, respectively. The transcriptional regulators will be activated once these signals reach a threshold (Venturi, 2006). The QS signals of *P. aeruginosa* have been detected in the lungs of infected cystic fibrosis patients (Singh et al., 2000; Favre-Bonté et al., 2002). Furthermore, a striking altered structure of biofilms and reduced virulence were observed in *P. aeruginosa* mutants that showed QS deficit (Davies et al., 1998; Pearson et al., 2000). A *lasI* mutant formed flat, abnormal and undifferentiated biofilms which were significantly different from the wild-type biofilms (Davies et al., 1998). Thus, the QS is being regarded as an attractive target for developing new therapeutics.

There are basically two strategies for interfering with the Gram-negative QS systems, known as enzyme degradation and the small molecules binding (Uroz et al., 2009). The latter has been extensively investigated by employing AHL analogs to bind the QS receptor site (Galloway et al., 2011). Due to the regulatory hierarchy of *P. aeruginosa* QS systems, the vast studies have focused on LasR (Welsh et al., 2015). For example, Geske (Geske et al., 2008) has synthesized a series of small molecules capable of sequestering OdDHL, effectively quenching the Las circuit. Compounds that specifically target Rhl are scarce. The synthetic AHL analogs mCTL and mBTL have been reported to act as RhlR antagonists that strongly inhibit pyocyanin production (O'Loughlin et al., 2013). Recently, more efforts have been aimed at screening antagonists of the *pqs* system (Storz et al., 2012; Lu et al., 2013). Though these synthetic AHLs show potential as anti-QS agents, production costs and success rates make drugs from natural products preferable (Kong et al., 2009). Fungi are renowned sources of natural products with an array of biological activities, such as antioxidant, antiviral, cytotoxic, and antibacterial (Kandasamy et al., 2015; Zhang S. P. et al., 2016). Fungi coexist with bacteria but lack active immune systems (Rasmussen et al., 2005). They instead rely on chemical defense mechanisms (Rasmussen et al., 2005). Recently, a series of anti-QS compounds such as penicillic acid (Wang et al., 2011), ω-hydroxyemodin, emodic acid, and (+)-2′S-isorhodoptilometrin (Figueroa et al., 2014) have been isolated from *Penicillium* sp. Thus, the use of fungi to control pathogenic bacteria is believed to be a renewable approach. Phyllosphere should deserve a special attention because it is a vital habitat for QS quenching fungi (Lindow and Brandl, 2003). Notably, fungi have the capacity to reside within the same ecological niche as their pathogenic counterparts. This could protect their host plants effectively (Osono, 2006). In China, *Orychophragmus violaceus* has been used as a medicinal and edible plant for centuries (Zhan et al., 2016). However, literature about the bioactive potential of *O. violaceus* is limited (Ping, 2009). Furthermore, phyllosphere fungi derived from *O. violaceus* have not been reported. In order to screen new QS inhibitors, the aim of this work was to explore phyllosphere fungi that could produce antivirulence compounds and study the influence of their metabolites on the QS mediated biofilms of *P. aeruginosa* PAO1. Finally, the principal antivirulence compounds contained in the metabolites of *Plectosphaerella cucumerina* were also investigated.

## Materials and methods

### Isolation and identification of the phyllosphere fungi

Healthy leaves were randomly collected from *O. violaceus* at different locations grown in Nanjing University of Science and Technology, Nanjing, China. To isolate the phyllosphere fungi, mixed leaves (10 g) were placed in sterile flasks containing 30 mL of potassium phosphate buffer (PBS, 0.1 M, pH 7.0), and sonicated for 10 min at 40 KHz power level at 20°C using a ultrasonic cleaner (Lv et al., 2013). The mixture was then vortexed for 10 min and the resulting suspension was serially diluted and spread onto Czapek-dox agar plates (0.3% NaNO_3_, 0.1% K_2_HPO_4_, 0.05% MgSO_4_·7H_2_O, 0.05% KCl, 0.001% FeSO_4_, 3% sucrose, and 1.5% agar; Han et al., 2016) supplemented with streptomycin sulfate (250 mg L^−1^) and ampicillin (250 mg L^−1^) to inhibit bacterial growth. The plates were incubated at 24°C and the purified isolates were stored at −80°C for further use. Colony morphology was determined after 14 days of incubation on Potato Dextrose Agar (20% potato, 2% dextrose, 2% agar, PDA; Bai and Shaner, 1996) at 24°C in darkness. Fungal internal transcribed spacer (ITS) region (ITS1, 5.8S rRNA and ITS4) (Figueroa et al., 2014; Zhang T. et al., 2016), was amplified using primers ITS-1 (5′-TCCGTAGGTGAACCTGCGG-3′) and ITS-4 (5′-TCCTCCGCTTATTGATATGC-3′; Zhang T. et al., 2016). PCR were assayed in 25 μL of mixtures with 2.5 μL of 10 × buffer (with Mg^2+^) (Sangon Biotech, China), 1 μL of dNTP (2.5 mM) (Sangon Biotech, China), 0.2 μL of enzymes (Sangon Biotech, China), 1 μL of primers and 0.5 μL of DNA. The applied temperature cycle was: initial denaturation at 95°C for 5 min, 30 cycles of 45 s of denaturation at 95°C, 45 s of annealing at 55°C, 1 min of extension at 72°C, and a final extension for 10 min at 72°C. PCR products were purified employing a SanPrep extraction kit (Sangon Biotech, China). The sequencing reaction was assayed through the Applied Biosystems 3730XL automated sequencer system. The sequences were compared with Genbank databases followed by sequence alignment. Phylogenetic trees were performed by neighbor-joining algorithm, using MEGA version 4.1 (Tempe, AZ, USA).

### Indicator strains and growth conditions

The bacterial strains such as *Agrobacterium tumefaciens* KYC55 (pJZ372, pJZ 384, pJZ410) and *P. aeruginosa* PAO1 (wild type) were used in this study based on their QS dependent phenotypes. *P. aeruginosa* PAO1 was kindly provided by Prof. Qianhong Gong, Ocean University of China (Qingdao, China). *A. tumefaciens* KYC55 was kindly presented by Prof. Jun Zhu, University of Pennsylvania (Philadelphia, USA). *A. tumefaciens* KYC55 was propagated in Luria Bertani medium (LB, Sangon Biotech, China) containing 1% tryptone, 0.5% yeast extract, and 1% NaCl (Wu et al., 2015) without antibiotics at 30°C. *P. aeruginosa* PAO1 was cultivated in LB at 37°C unless otherwise specified.

### Preparation of extract of *P. cucumerina*

*P. cucumerina* was cultivated in PDA medium for 7 days until the mycelium was widespread over the plate. Two hundred and fifty milliliters Erlenmeyer flasks containing 100 mL of Fungus No. 2 medium (2% sorbitol, 2% maltose, 1% glutamine, 1% glucose, 0.3% yeast extract, 0.05% tryptophan, 0.05% KH_2_PO_4_, 0.03% MgSO_4_, pH 6.4, autoclaved at 121°C for 15 min; Xin et al., 2007) were inoculated with the fungal strain. The flasks were incubated at 28°C at 140 rpm for 15 d. After incubation, the fungal biomass and the fermentation broth were separated by centrifugation at 10,000 rpm for 15 min. The fungal biomass was lyophilized using a lyophilizer (LGJ-25C, Four Ring Science Instrument Plant Beijing Co., Ltd., Beijing, China) at −50°C for 15 h. The fungal biomass and the fermentation broth were extracted with the same volume of ethyl acetate (EtOAc). The solvent was pooled and eliminated under reduced pressure and residues were dissolved in methanol (MeOH) and stored at −20°C.

### The minimum inhibitory concentration and growth measurement

*P. cucumerina* extract was tested against the selected bacterial strains to determine their minimum inhibitory concentration (MIC) according to Clinical and Laboratory Standards Institute (CLSI) (2015) with an inoculum of about 1–5 × 10^5^ CFU mL^−1^. Serial two-fold dilution (0.16–10 mg mL^−1^) of extract was performed in Müller-Hinton broth (Sangon Biotech, China). The MIC was the lowest concentration of extract that inhibited visible cell growth. For growth measurement, overnight cultures of *P. aeruginosa* PAO1 were subcultured into LB medium to achieve OD_620_ of 0.05. The cultures were then supplemented with extract with varying concentrations and incubated at 37°C at 150 rpm for 15 h. In this study, the extract was firstly set into a high concentration with an amount of MeOH. The extract was then diluted into different concentrations with MeOH. All test extracts with the varying concentrations were supplemented with the same volume. The same amount of distilled water (0) and MeOH served as negative controls. Growth was evaluated by measuring OD_620_ using a microplate reader (Biotek Elx800, USA) in triplicate.

### Analysis of the AHLs produced by *P. aeruginosa*

To assess the inhibitory effect of *P. cucumerina* extract on the levels of C4-HSL and 3-oxo-C12-HSL produced by *P. aeruginosa*, the overnight cultures of *P. aeruginosa* PAO1 were diluted 1:1,000 into 50 mL of LB and incubated at 37°C at 200 rpm for 48 h. The extract was added as treated group while the same amount of water and MeOH were used in control groups. After incubation, cells were separated from culture fluids by 15-min centrifugation (12,000 rpm, 4°C). Cell-free culture fluids were extracted with an equal volume of acidified ethyl acetate (0.5% formic acid) for three times. The solvent was pooled and eliminated under reduced pressure at 35°C and residues were dissolved in MeOH and stored at −20°C prior to analysis. The levels of AHLs were determined using LC-MS/MS as described previously (Morin et al., 2003). AHLs were analyzed using HPLC system (SHIMADZU) equipped with a Inertisl-C18 column (250 × 4.6 mm, 5 μm; Shimadzu, Tokyo, Japan). Mobile phase A was formic acid (0.1%) and ammonium formate (5.0 mM) in water. Mobile phase B was MeOH. The flow rate was set as 0.4 mL min^−1^. The injection volume was 10.0 μL. The gradient was set as: 1–5 min, 20–50% B; 5–20 min, 50–90% B. The eluates were determined by a ThermoFinnigan LCQ Classic system (San Jose, CA) using the positive mode. The nebulizer was set as 15 psi. The drying gas was set as 7 mL min^−1^ and the temperature was maintained at 300°C. Full-scan MS was from *m/z* 100 to 1,000. The peaks corresponding to C4-HSL and 3-oxo-C12-HSL were identified according to their fragment MS ions and the retention time of commercial standards (Sigma-Aldrich, USA) for each AHL. The area of the ion *m/z* 102 was selected to quantify each AHL due to its specificity and better signal-to-noise ratio. The extracted ion chromatograms were employed for area calculation. This is a relative quantification approach for AHLs without the need of standard curve. Data were normalized to the level of the water control for relative quantification.

### Anti-biofilm formation assay

*P. aeruginosa* PAO1 biofilms were quantified based on the tube method (Panda et al., 2016). Overnight, *P. aeruginosa* cultures were diluted 1:50 into 200 μL of Trypticase Soytone broth (TSB, 1.7% tryptone, 0.3% soy protone, 0.25% glucose, 0.5% NaCl, 0.25% KH_2_PO_4_, pH 7.2, OD_620_ ≈ 0.1; Cycoń et al., 2016) with varying concentrations of extract. Distilled water and MeOH served as negative controls. The tubes were incubated at 37°C for 24 h without agitation. After incubation, excess broth and the planktonic cells were removed. For quantification of the total biofilm biomass, the tubes were washed with PBS (pH 7.2) and then dried at 60°C for 30 min. The biofilms were stained with 200 μL of 0.1% crystal violet for 15 min and then rinsed off by distilled water and bounded crystal violet was re-solubilized in 100% ethanol. The OD was determined at 570 nm using a microplate reader (Biotek Elx800, USA). The biofilm biomass was normalized to the level of the water control for relative quantification.

Subsequently, the effect of extract on exopolysaccharide (EPS) was determined using the sonication method (Liu et al., 2007; Gong et al., 2009; Kim and Park, 2013). Briefly, *P. aeruginosa* PAO1 was incubated in AB medium (300 mM NaCl, 50 mM MgSO_4_, 10 mM potassium phosphate, 0.2% vitamin-free casamino acids, 1% glucose, 1 mM L-arginine; Kim and Park, 2013) in the absence and presence of extract (0.25–1 mg mL^−1^) at 37°C for 24 h without agitation. The cultures were then centrifuged at 12,000 rpm for 15 min to remove the cells. The cell pellets were resuspended in 10 mL of KCl (0.01 M) and then sonicated with a sonicator (YJ96-II, Ningbo Scientz Biotechnology Co., Ltd., Ningbo, China) with four cycles of 5 s on and 5 s pauses at 3.5 Hz power level. The sonicated suspension was centrifuged (12,000 rpm, 4°C) once again for 20 min. The supernatant was filtered through a 0.22-μm filter (Millipore, Fisher Scientific). For carbohydrate quantification, 50 μL of the filtrate coupled with 150 μL of concentrated sulfuric acid were added into a 96-well polystyrene microtiter plate and incubation at room temperature for 30 min. The mixture was added with 5% phenol and incubated at 90°C in the water bath for 5 min. The amount of carbohydrate was quantified by measuring OD_490_ and the relative EPS production was calculated.

### Biofilm disruption assay

The overnight cultures of *P. aeruginosa* PAO1 were diluted 1:50 into 200 μL of TSB, and incubated statically in 1.5-mL tube at 37°C for 24 h. The biofilm was then treated with varying concentration of extract for 24 h. After incubation, both the culture medium and the planktonic cells were removed. For quantification of the total biofilm biomass, the tubes were washed with PBS for three times and then dried at 60°C for 30 min. The biofilm biomass was determined employing the crystal violet staining method as described above.

### Microscopy analysis

For the biofilm inhibition assay, the 96-well, round-bottom, sterile polystyrene microtiter plate (Coming Costar, Ltd., New York, USA) with circular glass slides (diameter of 14 mm) and overnight cultures of *P. aeruginosa* PAO1 were supplemented with extract and incubated at 37°C for 24 h without agitation. Distilled water and MeOH served as negative controls. For quantification of the total biofilm biomass, the glass slides were washed with PBS for three times and then dried at 60°C. Biofilms were stained with 0.1% crystal violet or 0.01% acridine orange for 15 min. After which, biofilms were observed under light microscope (Nikon 80i, Japan) and fluorescence microscope (Nikon 80i, Japan), respectively.

For the biofilm disruption assay, after biofilms formed, the wells were supplemented with or without extract and incubated for 24 h. The biofilms that adhered to the glass slides were then stained using acridine orange for 15 min and visualized under fluorescence microscope. For SEM analysis, slides coated with biofilms were fixed using 2.5% glutaraldehyde and dehydrated with ethanol (50, 70, 80, 90, and 100%). The slides were subsequently dried, gold coated and observed under SEM (JSM6360, JEOL, Tokyo, Japan).

### Effect of extract on *P. aeruginosa* virulence factors

Protease activity was determined according to Wu et al. (2016) using the azocaserin method. Azocaserin is a suitable substrate for the measurement of protease activity (Hazen et al., 1965). During digestion, the colored components are formed and soluble in trichloroacetic acid. After removal of the undigested substrate, the color, which is proportional to the proteolytic activity of the enzyme, can be measured by a microplate reader (Hazen et al., 1965). Briefly, 150 μL of sterile supernatant of *P. aeruginosa* PAO1 and 250 μL of 2% azocasein (Sangon Biotech, China) in 50 mM Tris-HCl were incubated at 37°C for 4 h. The undigested substrate was precipitated with trichloroacetic acid (10%, 1.2 mL) for 20 min and then centrifuged at 12,000 rpm for 10 min, after which the supernatant was supplemented with 1.4 mL of 1 M NaOH. The absorbance was then measured at 440 nm.

Elastase activity was measured as described previously (Ohman et al., 1980). Briefly, 900 μL of Elastin congo red (ECR) buffer (1 mM CaCl_2_, 100 mM Tris, pH 7.5) with 20 mg ECR was mixed with 100 μL of culture supernatant and incubated for 3 h at 37°C. Absorbance of the supernatant was measured at 495 nm after removing the insoluable ECR by centrifugation at 12,000 rpm for 10 min.

Quantification of pyocyanin was performed as previously described with minor modification (O'Loughlin et al., 2013). *P. aeruginosa* strains were propagated in a 2-mL Pseudomonas broth containing 2% bacto peptone, 0.14% MgCl_2_, 1% K_2_SO_4_, and 1% glycerol and were incubated at 37°C at 150 rpm for 17 h. Cells were separated from culture fluids by 15-min centrifugation at 12,000 rpm. Cell-free culture fluids were used to quantify pyocyanin by measuring OD_695_.

Rhamnolipids were assayed using the orcinol method with minor modification (Kim et al., 2015). Culture supernatant with the volume of 300 μL was extracted with 600 μL of diethyl ether. The organic phase was collected and evaporated to dryness under reduced pressure at 35°C and then added with 100 μL of deionized water. Orcinol solution with the volume of 900 μL (0.19%, Sigma-Aldrich) in 53% H_2_SO_4_ was mixed with 100 μL of each sample. The mixture was heated at 80°C for 30 min, and then cooled at room temperature for 15 min. Rhamnolipids concentration was determined at 421 nm.

Quantification of alginate was assayed as described previously (Owlia et al., 2007). Sterile-filtered supernatant (70 μL) of *P. aeruginosa* PAO1 was mixed with 600 μL of boric acid/H_2_SO_4_ (4:1, v/v). The mixture was vortexed and supplemented with 20 μL of 0.2% carbazole solution. The mixture was vortexed and incubated at 55°C for 30 min. The OD value was determined at 530 nm.

Swimming and swarming motilities were assayed according to Sheng et al. (2015). For swimming, 3 μL of overnight cultures of *P. aeruginosa* PAO1 were inoculated in the swimming agar medium (1% tryptone, 0.5% NaCl, 0.3% agar) in the presence or absence of extract. For swarming motility, 5 μL of overnight cultures were inoculated in the swarming agar medium containing 1% tryptone, 0.5% NaCl, 0.3% agar, and 0.5% glucose. Plates were incubated at 37°C for 24 h.

### Thermal stability

*P. cucumerina* extract (1 mg mL^−1^) was exposed to a rising temperature gradient for 10 min (30, 50, 70, and 95°C). The stability of *P. cucumerina* extract was evaluated by an anti-pyocyanin assay as described above. Distilled water and MeOH served as negative controls. Quantification of pyocyanin was performed by measuring OD_695_.

### LC-MS/MS analysis of the extract

The *P. cucumerina* extract was analyzed using HPLC system (SHIMADZU) equipped with a XB-C18 column (100 × 2.1 mm, 3 μm; Welch Ultimate, Shanghai, China). The gradient was set as: 1–5 min, 5–20% B; 5–8 min, 20–40% B; 8–20 min, 40–100% B; 20–25, 100–100% B; 25–30 min, 100–5% B. Detection wavelength was 212 nm. For MS system, the nebulizer was set as 55 psi. The drying gas was set as 15 L min^−1^ and the temperature was maintained at 500°C. Full-scan MS was from *m/z* 100 to 1,000. Further verification and quantitation of patulin and emodin was performed by LC-MS/MS using their corresponding standards (Sangon Biotech, China). Electrospray ionization was conducted in the negative mode under the same conditions as mentioned above.

### Effect of patulin and emodin on *P. aeruginosa* biofilms and virulence factors

Furthermore, pure compounds of patulin (25 and 50 μg mL^−1^) and emodin (25 and 50 μg mL^−1^) were added to the growth medium to analyze their activities on *P. aeruginosa* virulence factors and biofilms. In this assay, patulin was dissolved in distilled water while emodin was dissolved in MeOH. Biofilms, pyocyanin, protease, elastase, and swimming motility were assayed as mentioned above.

### Statistical analysis

Data were expressed as mean values ± SD. The Levene's test for homogeneity of variance was applied to assess the equality of variances for all the variables before variance analysis. Statistically difference was determined by Levene's test coupled with ANOVA followed by Tukey–Kramer test. Statistics were performed using SPSS 18.0 software (SPSS, Inc., Chicago, IL, USA). *P* ≤ 0.05 was considered as significant.

## Results

### Identification of the phyllosphere fungus

**Figure 1:**
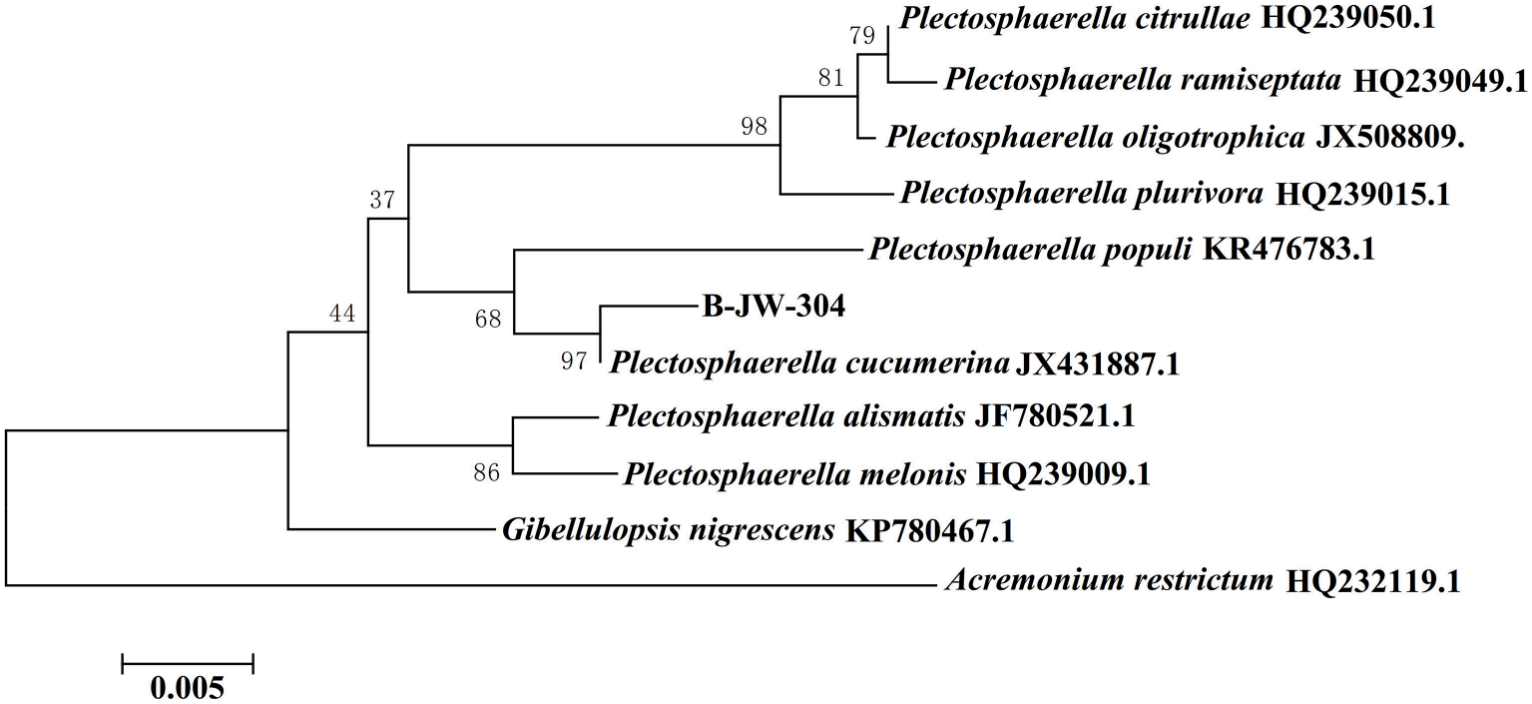
**Evolutionary relationships between *P. cucumerina* B-JW-304 with another 10 related strains**.

A total of 41 fungi were isolated from the healthy leaves of *O. violaceus* based on their morphological characteristics. The anti-QS capacity was preliminarily screened using *A. tumefaciens* KYC55 as reporter strain. The *A. tumefaciens* KYC55 generates the AHL receptor TraR, which can sense exogenous AHLs. This anti-QS capacity was determined by comparing the competitive binding of the AHL molecules of *A. tumefaciens* and fungal extract to the AHL receptors. The competition was evaluated colormetrically. Among 41 isolated fungi, 7 kinds of fungi showed anti-QS capacities against *A. tumefaciens* KYC55 (Figure S1). However, only one fungus coded “B-JW-304” was selected for further study due to its potent antivirulence and anti-biofilm capacities against *P. aeruginosa* PAO1. No antivirulence and anti-biofilm capacities were observed in the other 6 fungi. Colonies of this fungus on PDA were slimy, flat, appressed, with sparse aerial mycelium (Figure S2a). Aerial mycelium varied from buff to salmon pink. Mycelium branched, septate, with abundant anastomosis, forming hyphal coils (Figure S2b). Conidia hyaline, smooth, ellipsoid tapering to rounded ends (Figures S2c,d). Using molecular studies (based on ITS1-5.8S-ITS4), B-JW-304 was identified as *P. cucumerina* (Figure 1). The 18S rDNA sequence was deposited in Genbank under the accession number KX822030. *P. cucumerina* is a well-known pathogen that could cause fruit rot (Carlucci et al., 2012). At present, most reports about *P. cucumerina* are associated with its pathogenic effect and biological control (Sanchez-Vallet et al., 2010; Gamir et al., 2012). However, literature about the secondary metabolites secreted by *P. cucumerina* is scarce. Therefore, it is valuable to investigate the metabolites and evaluate their bioactive capacities from *P. cucumerina*.

### Determination of MIC of *P. cucumerina* extract

**Figure 2:**
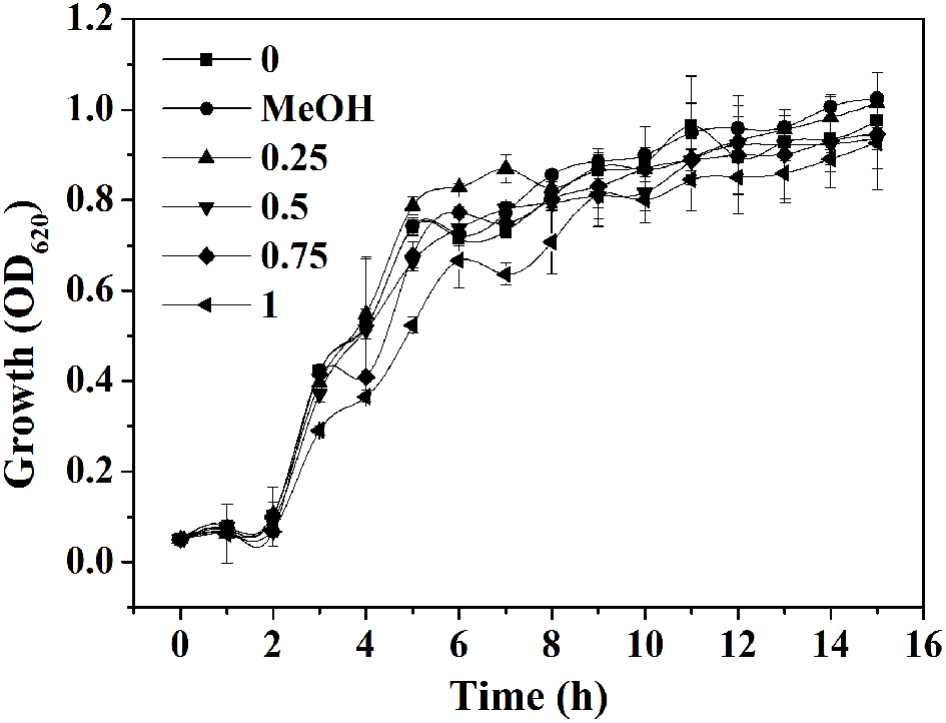
**Effect of *P. cucumerina* extract on *P. aeruginosa* PAO1 growth**. Growth at different concentrations of extracts (0.25, 0.5, 0.75, and 1 mg mL^−1^) for 15 h in tube. Distilled water (0) and MeOH served as negative controls. Error bars indicated the standard deviations of three measurements.

The yield of EtOAc extract in terms of dry weight was 314.8 mg L^−1^ of the pooled extract from cells and supernatant. The MIC of extract against *P. aeruginosa* PAO1 was 1.25 mg mL^−1^. The growth profile of *P. aeruginosa* PAO1 was evaluated using *P. cucumerina* extract at sub-MIC concentrations for 15 h (Figure 2). Results showed that *P. cucumerina* extract treatment with the concentration of 0.25, 0.5, 0.75, and 1 mg mL^−1^ had no inhibitory effect on cell growth comparing with the water control during the stationary growth phase (Table S1). However, the growth was statistically different between the cultures with MeOH and 1 mg mL^−1^ of extract (*P* < 0.05) at 12 and 14 h while not statistically different at 13 and 15 h (*P* > 0.05; Table S2). Therefore, we speculated that the experimental error might lead to such difference. To further validate the data, the growth assay was performed again. The bacterial viable count was performed after incubation for 24 h. The reason for selecting 24 h was that most assays of this study were determined after incubation for 24 h. The results showed that extract with the concentration of 1 mg mL^−1^ had no inhibitory effect on *P. aeruginosa* growth comparing with the two negative controls (Figure S3).

### Effect of *P. cucumerina* extract on AHLs levels

**Figure 3:**
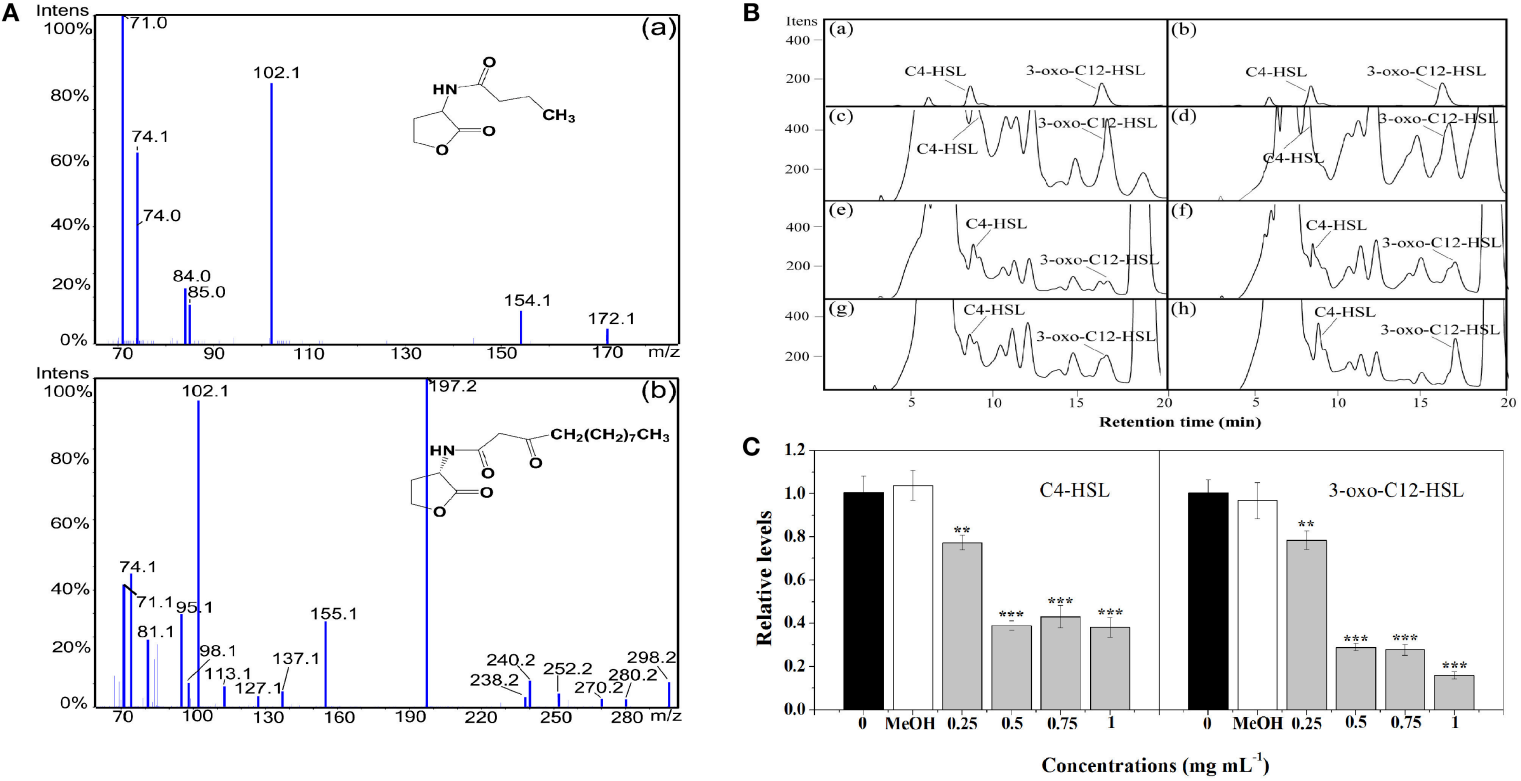
**Relative quantification of C4-HSL and 3-oxo-C12-HSL produced by *P. aeruginosa* using LC-MS/MS chromatograms. (A)** MS/MS spectrum of **(a)** C4-HSL and **(b)** 3-oxo-C12-HSL. **(B)** HPLC chromatograms of C4-HSL and 3-oxo-C12-HSL present in the supernatant from *P. aeruginosa* cultures supplemented with **(c)** distilled water, **(d)** MeOH, and **(e–h)** *P. cucumerina* extract (0.25, 0.5, 0.75, and 1 mg mL^−1^). **(a,b)** Represented the standards of C4-HSL and 3-oxo-C12-HSL. **(C)** Quantitative assessment of C4-HSL and 3-oxo-C12-HSL treated with *P. cucumerina* extract (0.25–1 mg mL^−1^). Distilled water (0) and MeOH served as negative controls. Error bars indicated the standard deviations of three measurements. Statistically difference was determined by ANOVA followed by Tukey–Kramer test. ^**^p < 0.01 vs. the water control (0). ^***^p < 0.001 vs. the water control (0).

In *P. aeruginosa*, the regulatory mechanism between *las* and *rhl* is clear. However, the molecular mechanism of *pqs* in coordinating virulence is currently unknown (Welsh et al., 2015). Therefore, in this study, the putative anti-QS activity of extract against *P. aeruginosa* was determined by quantitating the AHLs levels produced by this strain. LC-MS/MS analysis confirmed the presence of C4-HSL and 3-oxo-C12-HSL (Figures 3A,B). Treatment with *P. cucumerina* extract caused a decrease in both peaks and areas of C4-HSL and 3-oxo-C12-HSL as shown in the chromatograms (Figure 3B). The relative quantification of AHLs was presented in Figure 3C. *P. cucumerina* extract with the concentration of 0.25–1 mg mL^−1^ caused reduction of C4-HSL by ~22, 61, 57, and 62%, respectively comparing with the water control (Figure 3C). Further, a significant decrease was also observed in 3-oxo-C12-HSL after treatment with *P. cucumerina* extract from 0.25 to 1 mg mL^−1^ (Figure 3C). Overall, the data indicated that the anti-QS activity of *P. cucumerina* extract may be caused by interfering with the production of AHLs.

### Effect of *P. cucumerina* extract on biofilm development

**Figure 4:**
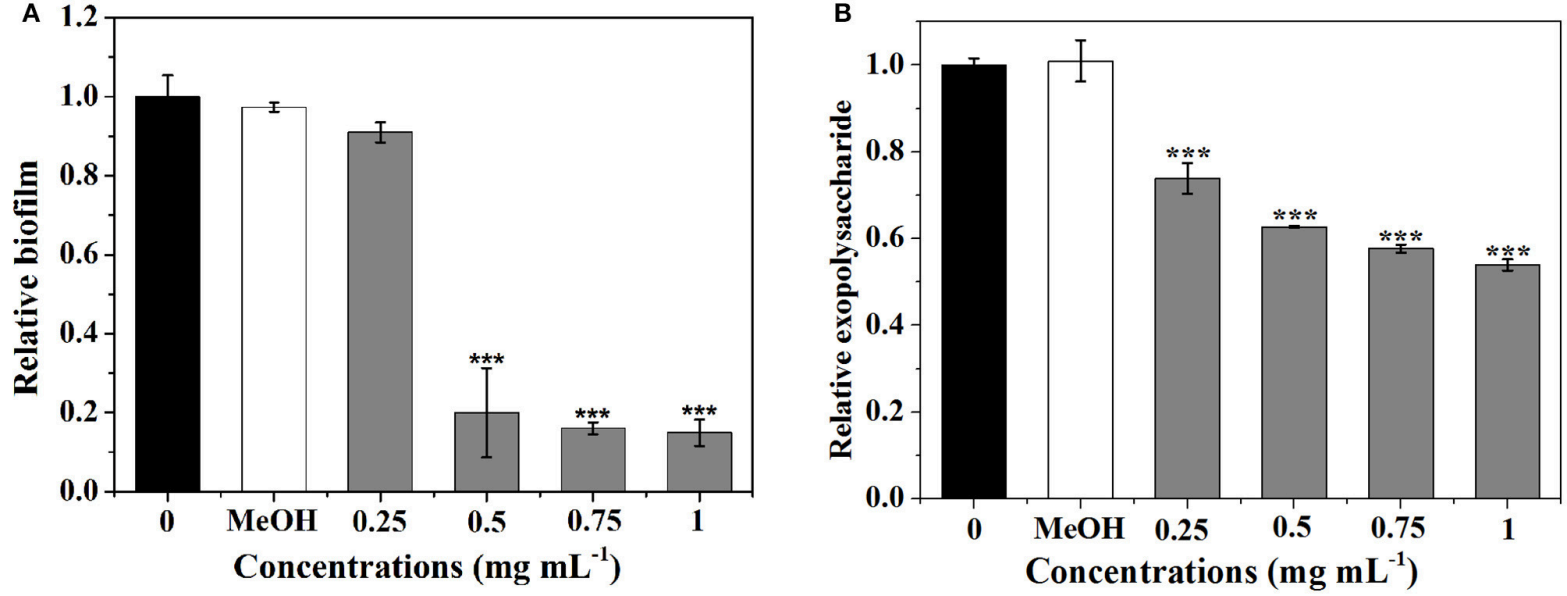
**Effects of *P. cucumerina* extract on biofilm development and exopolysaccharide production. (A)** Biofilm development and **(B)** exopolysaccharide production were quantified after 24 h of incubation by measuring at OD 570 and 490 nm, respectively. Error bars indicated the standard deviations of three measurements. Statistically difference was determined by ANOVA followed by Tukey–Kramer test. ^***^p < 0.001 compared with the water control (0).

As shown in Figure 4A, *P. cucumerina* extract inhibited biofilms formation in a concentration-dependent manner. Extract with the concentration of 0.5–1 mg mL^−1^ showed potent effects, reducing the biomass of PAO1 strain by ~80, 84, and 85%, respectively. MeOH showed no effect on the biofilm biomass of PAO1 comparing with the distilled water (0).

Since the formation of biofilm by *P. aeruginosa* is correlated with EPS (Kim and Park, 2013), the impact of extract on EPS production of *P. aeruginosa* PAO1 was evaluated. Figure 4B showed that extract exhibited concentration-dependent inhibitory activities against EPS and could reduce EPS production up to 46% at 1 mg mL^−1^.

**Figure 5:**
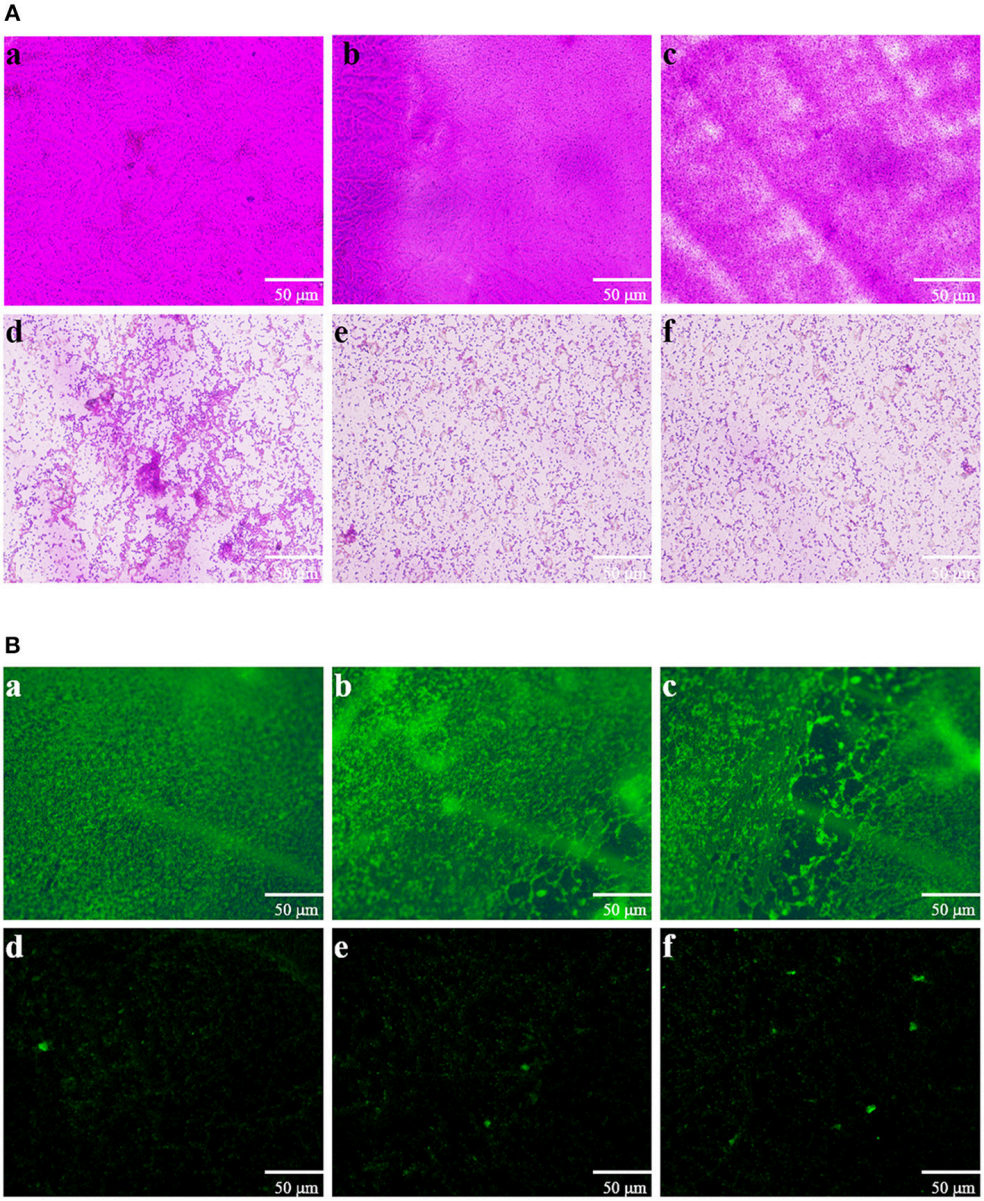
**Microscopic images of bacterial biofilms grown in the absence and presence of *P. cucumerina* extract. (A)** Light microscopic images and **(B)** fluorescence microscopic images of **(a)** distilled water, **(b)** MeOH, and **(c–f)** extract (0.25–1 mg mL^−1^) treated biofilms of *P. aeruginosa* PAO1.

Direct microscopic observations are recognized to provide useful information on biofilms. Thus, light microscopy and fluorescence microscope were employed. In the control experiment, where bacteria were not treated with extract, a thick coating of biofilms was detected (Figures 5Aa,b). As shown in Figure 5Ac, treatment with 0.25 mg mL-1 of extract showed no significant reduction in the microbial attachment. However, extract with the concentrations of 0.5, 0.75, and 1 mg mL^−1^ showed significant reduction in the microbial attachment to the glass surface compared to controls (Figures 5Ad–f). The above anti-biofilm activities were further confirmed by fluorescence microscope (Figure 5B). Fluorescence microscope images showed a scattered appearance of extract treated samples compared with the control. The cell clusters were rarely visible on account of poor cohesiveness.

### Effect of *P. cucumerina* extract on biofilm disruption

**Figure 6:**
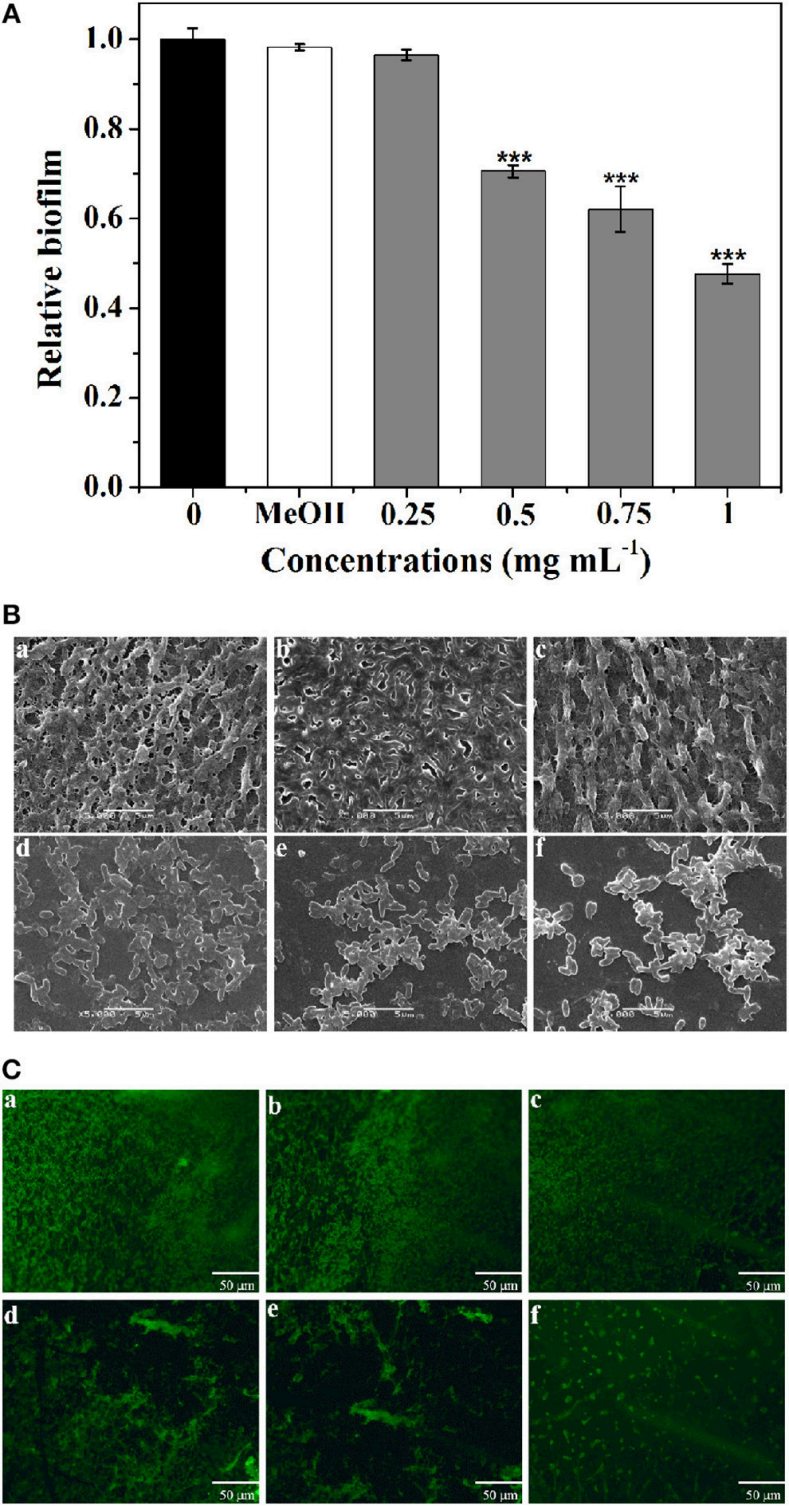
**Effect of *P. cucumerina* extract on biofilm disruption**. **(A)** Quantitative assessment of biofilm biomass disruption. Mean values of triplicate independent experiments and SD were shown. Statistically difference was determined by ANOVA followed by Tukey–Kramer test. ^***^p < 0.001 compared with the controls (0 and MeOH). **(B)** SEM images and **(C)** fluorescence microscopic images of **(a)** distilled water, **(b)** MeOH, and **(c–f)** extract (0.25–1 mg mL^−1^) treated biofilms of *P. aeruginosa* PAO1. Scale bars = 5 μm.

The biofilm disruption activity of *P. cucumerina* extract against PAO1 was measured employing a standard quantitative biofilm assay method (Ding et al., 2011). As shown in Figure 6A, the extract exhibited strong ability to disrupt the established biofilms when treated with 0.5–1 mg mL^−1^ extract. Biofilm biomass was reduced by ~52% in the presence of 1 mg mL^−1^ of extract.

Furthermore, SEM also showed the efficacy of extract as potent biofilm disrupter. The images showed a scattered appearance after treatment with extract when compared with the control colonization (Figure 6B). The cells associated with the surface were almost scattered and the integrity of the biofilms was limited. SEM, a destructive method, relies on rigid sample preparation and may generate pseudo-positive or unexpected results. To circumvent this problem, samples were assayed employing fluorescence microscope after staining with acridine orange (Figure 6C), and the results were similar to SEM analysis.

### Analysis of the production of virulence factors of *P. aeruginosa*

**Figure 7:**
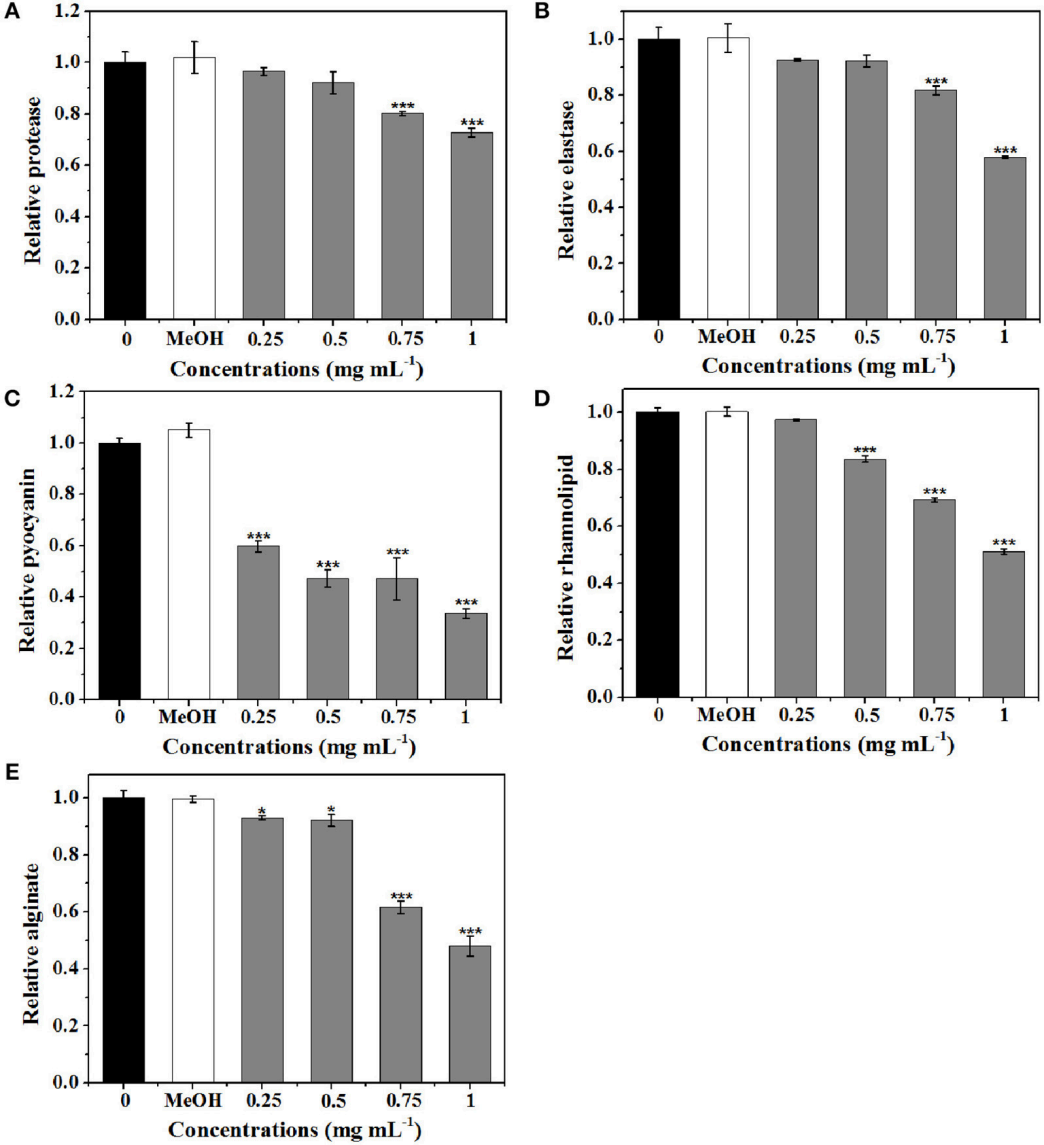
**Effects of *P. cucumerina* extract on virulence factors production in *P. aeruginosa* PAO1**. Activities of virulence factors at different extract concentrations (0.25, 0.5, 0.75, and 1 mg mL^−1^) were analyzed. Distilled water (0) and MeOH served as negative controls. **(A)** Protease activity. **(B)** Elastase activity. **(C)** Pyocyanin activity. **(D)** Rhamnolipid activity. **(E)** Alginate activity. Error bars indicated the standard deviations of three measurements. Statistically difference was determined by ANOVA followed by Tukey–Kramer test. ^*^p < 0.05 vs. the water control (0). ^***^p < 0.001 vs. the water control (0).

Five virulence factors (protease, elastase, pyocyanin, rhamnolipid, and alginate) of *P. aeruginosa* were analyzed to evaluate the effects of extract on virulence (Figure 7). As shown in Figure 7A, the expression level of protease was significantly suppressed by 0.75 and 1 mg mL^−1^ of extract. At 1 mg mL^−1^, 27.3% inhibition of protease activities was observed. Production of elastase by *P. aeruginosa* is one of the QS-dependent behaviors (Husain et al., 2015). As shown in Figure 7B, the reduction in elastase was concentration-dependent. Approximately 42% inhibition in elastase was detected after treatment with extract at 1 mg mL^−1^.

Pyocyanin is a vital factor for infection and biofilm formation in *P. aeruginosa* (Wu et al., 2016). Figure 7C showed a significantly decreased production of pyocyanin treated with extract. At 0.25 mg mL^−1^, there was a 40% decrease in pyocyanin production and at 1 mg mL^−1^, nearly 70% inhibition was observed. Furthermore, extract also exhibited concentration-dependent inhibitory activities against rhamnolipid and could reduce rhamnolipid production up to 50% at concentration of 1 mg mL^−1^ (Figure 7D).

As alginate is a constituent of the extracellular matrix of *P. aeruginosa* biofilms (Luo et al., 2016), the efficiency of extract to reduce the alginate production was determined. Results demonstrated that the alginate production was significantly reduced with increasing concentrations of extract. Approximately 52% reduction was observed when exposure to extract at 1 mg mL^−1^ (Figure 7E).

### Swarming and swimming inhibition assay

**Figure 8:**
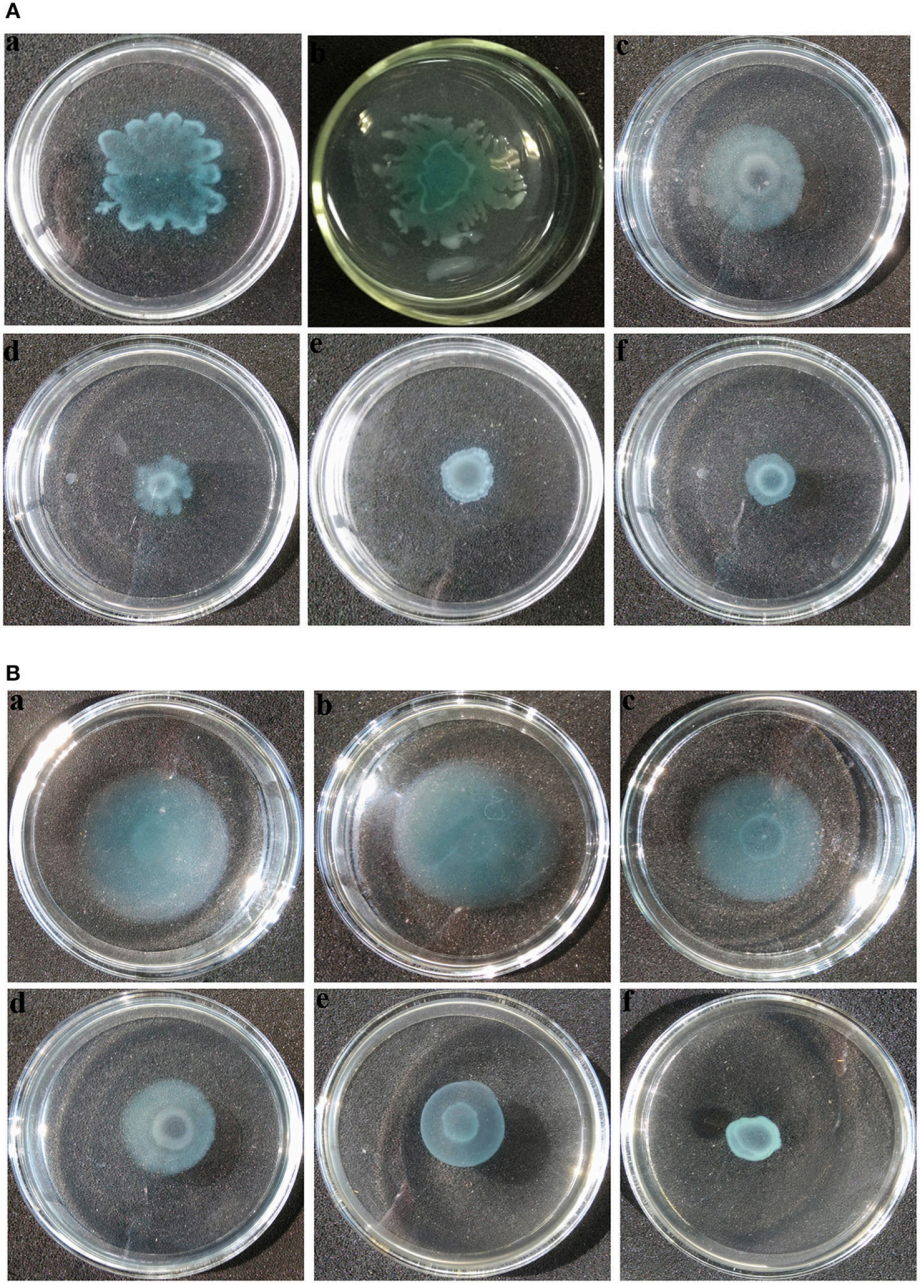
**Motility inhibition assays of *P. aeruginosa* PAO1. (A)** Swarming and **(B)** swimming motility assays were performed on plates containing different concentrations of agar in the absence or presence of *P. cucumerina* extract. **(a)**, treated with distilled water; **(b)**, treated with MeOH; **(c–f)**, treated with 0.25–1 mg mL^−1^ extract. The results shown were representative of results from three independent experiments.

Swarming and swimming motility play important roles in QS-mediated biofilm formation in uropathogens (Jones et al., 2004). Inhibition of swarming and swimming motility was observed at concentrations as low as 0.25 mg mL^−1^ of extract (Figures 8Ac,Bc). *P. aeruginosa* PAO1 exhibited swarming motility with a total swarming diameter of 33 mm. When treated with extract at concentrations ranging from 0.5 to 1 mg mL^−1^, bacteria could grow and form a colony in the center with a diameter not exceeding 12 mm, and the tendril formation was also observed to be significantly reduced (Figures 8Ad–f) when compared with the negative controls (Figures 8Aa,b). Furthermore, similar results were observed in swimming motility as well (Figures 8Ba–f).

### Thermal stability of *P. cucumerina* extract

In this study, thermal stability was measured by 10 min incubation of the extract at varying temperatures ranging from 30 to 95°C, and then the anti-pyocyanin capacity of *P. cucumerina* extract was determined. Results shown in Figure S4 demonstrated that the *P. cucumerina* extract was relatively thermal stable. More than 90% activity of the extract was remained even after being maintained at 95°C for 10 min when compared to heat treatment at 30°C.

### Potential bioactive compounds contained in *P. cucumerina* extract

**Figure 9:**
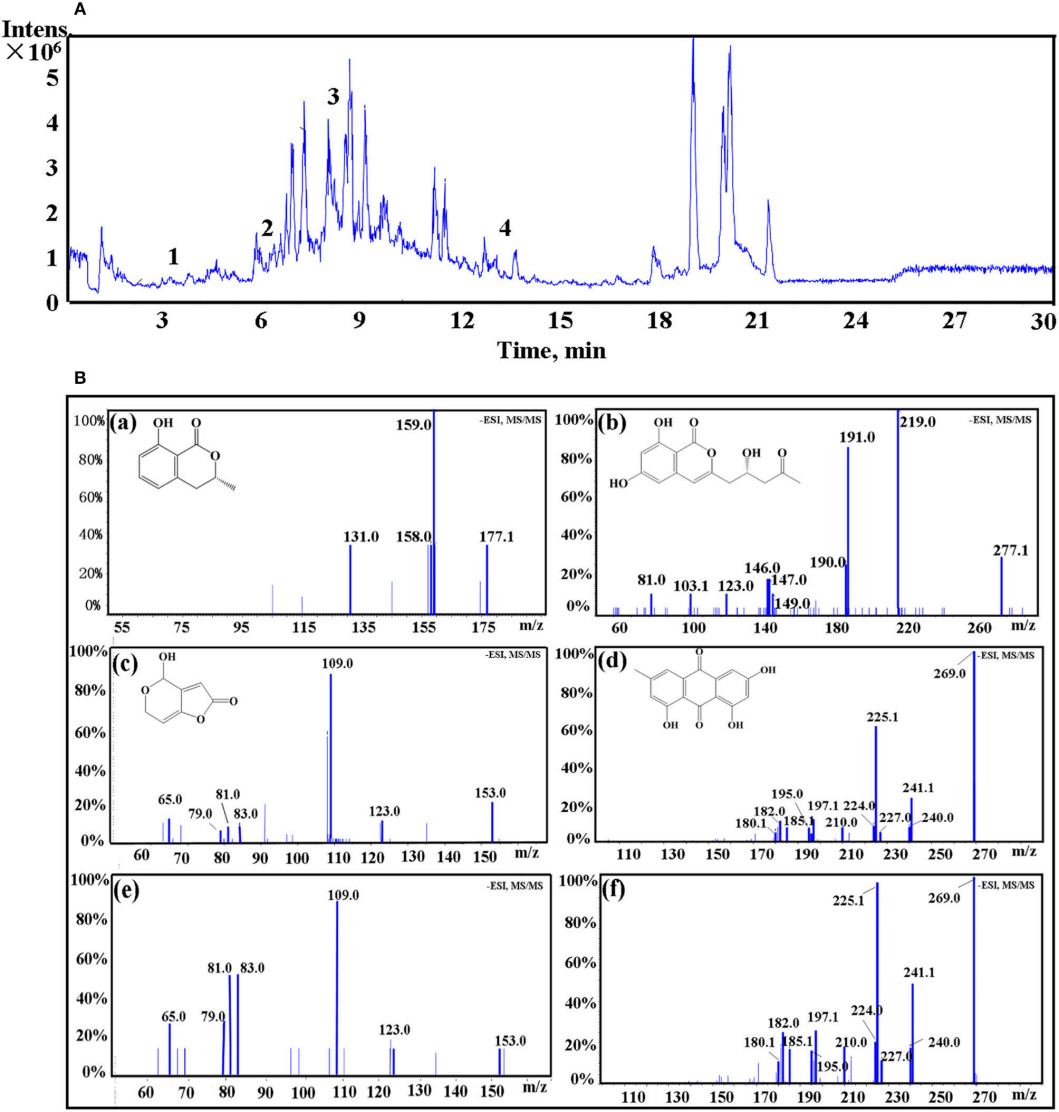
**(A)** The total ion chromatogram of *P. cucumerina* extract. Peak numbers correspond to chemical compounds presented in Table 1. **(B)** Spectra of ion fragments obtained from analysis of *P. cucumerina* extract and standard samples of emodin and patulin. Analysis performed by using ESI in negative ion mode. Ion fragments correspond to chemical compounds presented in Table 1. **(a)** MS/MS spectrum of mellein; **(b)** MS/MS spectrum of citreoisocoumarin; **(c)** MS/MS spectrum of patulin; **(d)** MS/MS spectrum of emodin; **(e)** MS/MS spectrum of a commercial standard patulin; **(f)** MS/MS spectrum of a commercial standard emodin.

LC-MS/MS was employed to identify potential bioactive compounds in *P. cucumerina* extract (Figure 9A). As shown in Table 1, four compounds were identified. The extract ion chromatogram at *m/z* 177.0556 showed peak eluting at 5.87 min. This peak displayed the fragment at *m/z* 159 (M-H-H_2_O) and 131 (M-H-CH_2_O_2_) (Figure 9Ba). The spectrum was indicative of mellein (Zhang X. et al., 2016). Compound **2** yielded a quasi-molecular ion [M-H]^−^ at *m/z* 277.0718 and fragment MS ions at *m/z* 219 (M-H-C_2_H_2_O_2_) and 191 (M-H-C_3_H_2_O_3_) (Figure 9Bb), being identified as citreoisocoumarin. These fragments are in accordance with the same profile described by one previous study (Rasmussen et al., 2010). The extract ion chromatogram at *m/z* 153.0194 in *P. cucumerina* extract showed a peak at 3.39 min (Table 1). This peak displayed the fragments at *m/z* 109 (M-H-CO_2_) and 81 (M-H-CH_2_O_3_) (Figure 9Bc). The spectrum data was consistent with that of patulin (Desmarchelier et al., 2011), which was further validated by a commercial standard patulin (Figure 9Be). Quantitation analysis showed that the production of patulin was 17.62 μg mL^−1^ of fungal extract (1 mg mL^−1^) and this concentration was comparable with the production of 5.5 μg mL^−1^ of patulin present in fungal broth (Figure S5A). The extract ion chromatogram at *m/z* 269.0454 showed a peak at 13.18 min. This peak displayed the fragments at *m/z* 241 (M-H-CO), 225 (M-H-CO_2_), and 197 (M-H-CH_2_O_3_) (Figure 9Bd). This spectrum was indicative of emodin (Song et al., 2008), which was also further validated by a commercial standard emodin (Figure 9Bf). Further, the production of emodin was quantified by 20.37 μg mL^−1^ of fungal extract (1 mg mL^−1^) and this concentration was comparable with the production of 6.4 μg mL^−1^ of emodin present in fungal broth (Figure S5B).

**Table 1:** **Mass spectrometric data of compounds 1–4 identified in EtOAc extract of *P. cucumerina* using HPLC Triple TOF MS/MS**.

**Peak**	**R_t_/min**	**Molecular formula**	**Tentative identification**	**Calculated [M−H]^−^**	**Measured [M−H]^−^**	**Error/ppm**	**MS^2^**
1	3.39	C_7_H_6_O_4_	Patulin	153.0193	153.0194	0.7	109, 81
2	5.87	C_10_H_10_O_3_	Mellein	177.0557	177.0556	−0.8	159, 131
3	7.94	C_14_H_14_O_6_	Citreoisocoumarin	277.0718	277.0718	0.1	219, 191
4	13.18	C_15_H_10_O_5_	Emodin	269.0456	269.0454	−0.6	241, 225, 197

### Effect of the four compounds on *P. aeruginosa* biofilms and virulence factors

**Figure 10:**
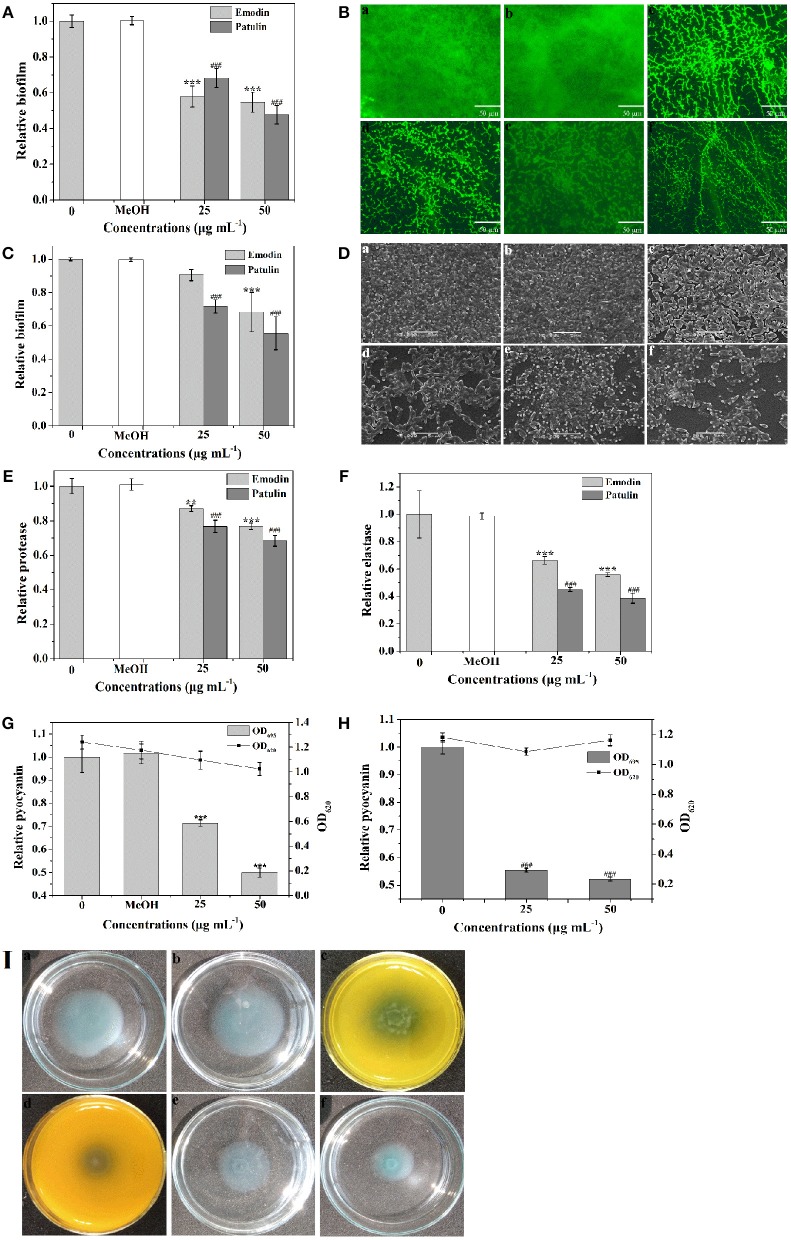
**Effects of emodin (25 and 50 μg mL^−1^) and patulin (25 and 50 μg mL^−1^) on biofilm development and virulence factors. (A)** Biofilm development was quantified after 24 h of incubation by measuring at OD 570. **(B)** Fluorescence microscopic images of **(a)** distilled water, **(b)** MeOH, **(c,d)** emodin (25 and 50 μg mL^−1^, respectively), and **(e,f)** patulin (25 and 50 μg mL^−1^, respectively) treated biofilms of *P. aeruginosa* PAO1. **(C)** Quantitative assessment of biofilm biomass disruption. **(D)** SEM images of **(a)** distilled water, **(b)** MeOH, **(c,d)** emodin (25 and 50 μg mL^−1^, respectively), and **(e,f)** patulin (25 and 50 μg mL^−1^, respectively) treated biofilms of *P. aeruginosa* PAO1. Scale bars = 5 μm. **(E)** Protease activity. **(F)** Elastase activity. **(G)** Effects of emodin on pyocyanin production of *P. aeruginosa* PAO1. **(H)** Effects of patulin on pyocyanin production of *P. aeruginosa* PAO1. **(I)** Swimming motility assays were performed on plates in the presence of **(a)** distilled water, **(b)** MeOH, **(c,d)** emodin (25 and 50 μg mL^−1^, respectively), and **(e,f)** patulin (25 and 50 μg mL^−1^, respectively). Error bars indicated the standard deviations of three measurements. Statistically difference was determined by ANOVA followed by Tukey–Kramer test. ^**^p < 0.01 vs. the water control (0). ^***^p < 0.001 vs. the controls (0 and MeOH). ^###^*p* < 0.001 vs. the control (0).

To screen the bioactive compounds responsible for the anti-biofilm and antivirulence activities of *P. cucumerina* extract, four identified compounds above were tested. Unfortunately, mellein and citreoisocoumarin showed no anti-biofilm and antivirulence activities (Figure S6). Patulin has been reported to inhibit QS-controlled gene expression in *P. aeruginosa* PAO1 (Rasmussen et al., 2005). Apart from gene expression inhibition, could patulin inhibit QS regulated phenomena such as biofilms and production of virulence factors or not? In this study, the MIC of patulin was 100 μg mL^−1^. *P. aeruginosa* PAO1 treated with 25 and 50 μg mL^−1^ of patulin showed 32 and 52% reduction in biofilm formation, respectively (Figure 10A). The above anti-biofilm activities were further confirmed by fluorescence microscope (Figures 10Be,f). A scattered appearance was observed when exposure to patulin. Patulin also owned strong ability to disperse the established biofilms. Approximately 44% reduction in biofilm biomass was detected after treatment with patulin at 50 μg mL^−1^ (Figure 10C). SEM also showed the efficacy of patulin as an excellent biofilm disrupter, and a scattered appearance was observed in treated samples compared with the control colonization (Figures 10De,f). This is the first report of the biofilm disruption activity of patulin. To check the antivirulence effect of patulin on *P. aeruginosa*, virulence factors including protease, elastase, pyocyanin, and swimming were assayed since production of these factors was recognized as an indicator of active QS. Treatment with 50 μg mL^−1^ of patulin resulted in 31, 62, and 48% inhibition of protease, elastase, and pyocyanin, respectively (Figures 10E,F,H) without affecting cell growth (Figure 10H). Motility behavior was assessed by the swimming motility assay. Treated cells of *P. aeruginosa* showed shorter diameters on agar plates compared to the untreated controls (Figure 10I).

Emodin was another component identified in *P. cucumerina* extract by LC-MS/MS. We presumed that emodin might be another principal component for anti-biofilm and antivirulence in *P. cucumerina* extract. To validate this hypothesis, emodin with concentrations of 25 and 50 μg mL^−1^ (MIC was 94 μg mL^−1^) was analyzed. Treatment with 50 μg mL^−1^ of emodin showed a 45% reduction in biofilm formation (Figure 10A). The above anti-biofilm activities of emodin were further confirmed by fluorescence microscope (Figures 10Bc,d) comparing with the negative controls (Figures 10Ba,b). Biofilm disruption assay demonstrated that emodin (50 μg mL^−1^) treatment led to a 32% reduction in biofilm biomass (Figure 10C). SEM also revealed that biofilm formation decreased with the concentration of 50 μg mL^−1^ of emodin as compared to the controls (Figure 10D). The treated bacteria were in the discrete form rather than in a group. In addition, *P. aeruginosa* dosed with 50 μg mL^−1^ of emodin resulted in 23, 44, and 50% inhibition of protease, elastase, and pyocyanin, respectively (Figures 10E–G). Emodin treatment with the concentration of 50 μg mL^−1^ had no significantly inhibitory effect on cell growth comparing with the MeOH control but significantly with the water control (Figure 10H). To further confirm the data, the growth assay was performed again. The results showed that emodin with the concentration of 50 μg mL^−1^ had no inhibitory effect on *P. aeruginosa* growth comparing with both the negative controls (Figure S7). Swimming motility was also significantly inhibited after treatment with 25 and 50 μg mL^−1^ of emodin (Figures 10Ic,d).

Overall, the observed inhibition of *P. aeruginosa* biofilm and virulence factors suggested that patulin and emodin might act as the principal active components in *P. cucumerina* extract.

## Discussion

It is reported that nearly 80% of human infections are induced by biofilms (Ricucci and Siqueira, 2010). Biofilms are microbial communities that are closely associated with antibiotic resistance (Michalska and Wolf, 2015; Vadekeetil et al., 2016). Therefore, the exploration of natural compounds that could attenuate pathogenicity rather than cell growth has aroused great attention. Previous studies demonstrated that fungal metabolites were able to interfere with QS communication and/or attenuate QS-associated processes including biofilm formation or virulence factors secretion (Rasmussen et al., 2005; Wang et al., 2011). Recently, some anti-QS compounds secreted by an endophytic fungus *Penicillium restrictum* have been reported (Figueroa et al., 2014). However, no literature about the phyllosphere fungi with anti-biofilm and anti-quorum sensing activities has been reported to the best of our knowledge. In this study, a phyllosphere fungus was isolated from *O. violaceus* and identified as *P. cucumerina*. The metabolites of *P. cucumerina* could effectively reduce the AHLs production, biofilm formation and virulence factors of *P. aeruginosa*. The metabolites could also disrupt the preformed biofilms.

It is known that *P. aeruginosa* uses AHLs as QS signal to coordinate the expression of disease-causing attributes, such as motility, virulence factors secretion, and biofilm formation (Chan et al., 2015). Biofilm persistence is attributed to a matrix made up of EPS, lipids, and proteins (Flemming and Wingender, 2010). EPS serves as a protective barrier that could block the entry of antibiotics into cells (Fux et al., 2005). EPS secretion of *P. aeruginosa* PAO1 is controlled by QS systems (Nadell et al., 2008). Presently, a remarkable decrease of AHLs and EPS was detected in *P. aeruginosa* PAO1 with extract treatment. Along with the anti-biofilm characteristic, we speculated that *P. cucumerina* extract might inhibit the biofilm formation via interfering with the synthesis of AHLs, then inhibiting the formation of EPS, which is vital for biofilm formation and stability.

An additional remarkable characteristic of extract is its capacity of disrupting the preformed biofilms. Several polysaccharides have been reported to trigger biofilms dispersal (Rendueles et al., 2013). Mechanisms in the biofilm disruption have been proposed, including cell death, induction of cellular motility, and matrix-degrading enzymes (Karatan and Watnick, 2009; Kaplan, 2010). In spite of researches on the mechanisms of action of anti-biofilm compounds, the precise mechanisms are yet to be clarified (Rendueles et al., 2013). Therefore, it is attractive to uncover the detailed anti-biofilm mechanisms of extract in the future.

*P. aeruginosa* produces diverse virulence factors which are under the control of QS (Wu et al., 2014). We investigated the production of protease, elastase, pyocyanin, rhamnolipid, and alginate in the presence of *P. cucumerina* extract, and found that production of these factors were significantly reduced by *P. cucumerina* extract. Additionally, the effect of *P. cucumerina* extract on *P. aeruginosa* PAO1 was further determined by performing assays for motility. The swimming and swarming motilities of *P. aeruginosa* PAO1 were effectively reduced in the presence of *P. cucumerina* extract.

To understand the bioactive component of *P. cucumerina* extract, four metabolites mellein, citreoisocoumarin, patulin, and emodin were identified by LC-MS/MS. Mellein is an anthraquinone mycotoxin that was produced by *Penicillium islandicum* (Kawai et al., 1984). In this study, mellein showed no antivirulence or anti-biofilm capacities. Recently, coumarin was reported to possess antivirulence and anti-biofilm capacities against *P. aeruginosa* (Gutiérrez-Barranquero et al., 2015). Citreoisocoumarin, one coumarin analog, showed no antivirulence or anti-biofilm capacities in this study. The results suggested that the changes to the structural motif can influence the antivirulence efficacy. Apart from mullein and citreoisocoumarin, patulin and emodin showed potent antivirulence and anti-biofilm capacities at concentrations of 25 and 50 μg mL^−1^. Quantification analysis showed that the production of patulin and emodin were 17.62 and 20.37 μg mL^−1^ of fungal extract (1 mg mL^−1^), respectively. The productions were lower than the concentrations (25 and 50 μg mL^−1^) used in this study. These results suggested that *P. cucumerina* extract may contain other antivirulence and anti-biofilm compounds besides patulin and emodin.

In conclusion, this study clearly demonstrated that *P. cucumerina* extract not only inhibits virulence factors and biofilm formation, but also disrupt the preformed biofilms of *P. aeruginosa* PAO1. This is the first report of an antivirulence and anti-biofilm *P. cucumerina* from the phyllosphere of *O. violaceus*. The LC-MS/MS analysis further confirmed two antivirulence compounds patulin and emodin. All in all, this study provides fascinating new pathways for screening antipathogenic agents from phyllosphere fungus. It is not doubt that other anti-biofilm and antivirulence compounds should be presented in *P. cucumerina*, we will try to isolate such compounds in the near future.

## Author contributions

JZ and AJ conceived and designed the experiments. JZ, SB, and TC performed the experiments. JZ, HC, RY, ML, and YF analyzed the data. JZ and AJ wrote the paper.

## Funding

This work was supported by the grants from the National High Technology Research and Development Program of China (863 Program) (2014AA022208), Six Talent Peaks Project in Jiangsu Province, and the Fundamental Research Funds for the Central Universities (30916011307), the National Natural Science Foundation of China (31170131).

### Conflict of interest statement

The authors declare that the research was conducted in the absence of any commercial or financial relationships that could be construed as a potential conflict of interest.

## Supplementary material

Table S1 ***p*-values of extract treatment compared with water control group using ANOVA Tukey–Kramer test**.

Table S2 ***p*-values of extract treatment compared with MeOH control group using ANOVA Tukey–Kramer test**.

Figure S1 **Anti-QS activities of 7 kinds of fungi using *A. tumefaciens* KYC55 as a reporter strain**. A white halo around the well against a blue background was an indication of anti-QS zone. Overnight cultures of KYC55 (1 mL, OD_620_ ≈ 0.7) were aliquoted into 100 mL of warm molten AT agar medium [1.05% K_2_HPO_4_, 0.45% KH_2_PO_4_, 0.02% MgSO_4_·7H_2_O, 0.0005% FeSO_4_·7H_2_O, 0.001% CaCl_2_, 0.0002% MnCl_2_, 0.2% (NH_4_)_2_SO_4_, 0.2% D-mannitol, 0.8% agar, pH 7.2, autoclaved at 115°C for 15 min] and supplemented with 200 μL X-gal (50 μg mL^−1^, Sigma-Aldrich, USA) and 5 μM N-(3-oxooctanoyl)-L-homoserine lactone (Sigma-Aldrich, USA) and then gently mixed. The mixture was poured immediately over the surface of a solidified AT agar plate as an overlay. Wells of 5 mm in diameter were made on each plate. Finally, the fungal extract (10 μL) was added to the wells and the plates were incubated at 30°C for 24 h.

Figure S2 **Colony morphology of *P. cucumerina* B-JW-304. (a)** Colony on PDA after 14 d at 24°C. **(b)** Hyphal coil. **(c,d)** Septate conidia. Scale bars = 100 μm.

Figure S3 **Effects of *P. cucumerina* extract (1 mg mL^−1^) on *P. aeruginosa* PAO1 growth. (A)** Bacterial colonies of *P. aeruginosa* PAO1 (10^7^fold dilution) after incubation for 24 h. **(a)** Distilled water control; **(b)** MeOH control; **(c)** *P. cucumerina* extract (1 mg mL^−1^) treatment. **(B)** Bacterial colonies number of *P. aeruginosa* PAO1 after incubation for 24 h with (0) distilled water, MeOH and 1 mg mL^−1^ of extract treatment. Error bars indicated the standard deviations of three measurements.

Figure S4 **Activity analysis of *P. cucumerina* extract that was pretreated with heat (30–95°C) by using pyocyanin as evaluation index**. Error bars indicated the standard deviations of three measurements. ^***^*p* < 0.001 vs. the control (0).

Figure S5 **Standard curves targeting patulin (A)** and emodin **(B)** for quantification.

Figure S6 **Anti-pyocyanin (A)** and anti-biofilm **(B)** activities of mullein and citreoisocoumarin. Error bars indicated the standard deviations of three measurements.

Figure S7 **Effect of emodin on *P. aeruginosa* PAO1 growth**. Growth at concentrations of emodin (25 and 50 μg mL^−1^) for 17 h in tube. Distilled water (0) and MeOH served as negative controls. Error bars indicated the standard deviations of three measurements.
