# Supplementary material for: Anti-Biofilm and Antivirulence Activities of Metabolites from Plectosphaerella cucumerina against Pseudomonas aeruginosa
Source: Front Microbiol. 2017 May 3;8:769. doi: 10.3389/fmicb.2017.00769 (PMC5413567; doi:10.3389/fmicb.2017.00769)
Supplement: Table S1 — p-values of extract treatment compared with water control group using ANOVA Tukey–Kramer test. [file Table1.DOCX]

**Table S1** *p*-values of extract treatment compared with water control group.

| Time (h) | *p* value |
| --- | --- |
| 12 | 0.338 |
| 13 | 0.267 |
| 14 | 0.469 |
| 15 | 0.640 |
