# Supplementary figures and images for: Anti-Biofilm and Antivirulence Activities of Metabolites from Plectosphaerella cucumerina against Pseudomonas aeruginosa
Source: Front Microbiol. 2017 May 3;8:769. doi: 10.3389/fmicb.2017.00769 (PMC5413567; doi:10.3389/fmicb.2017.00769)

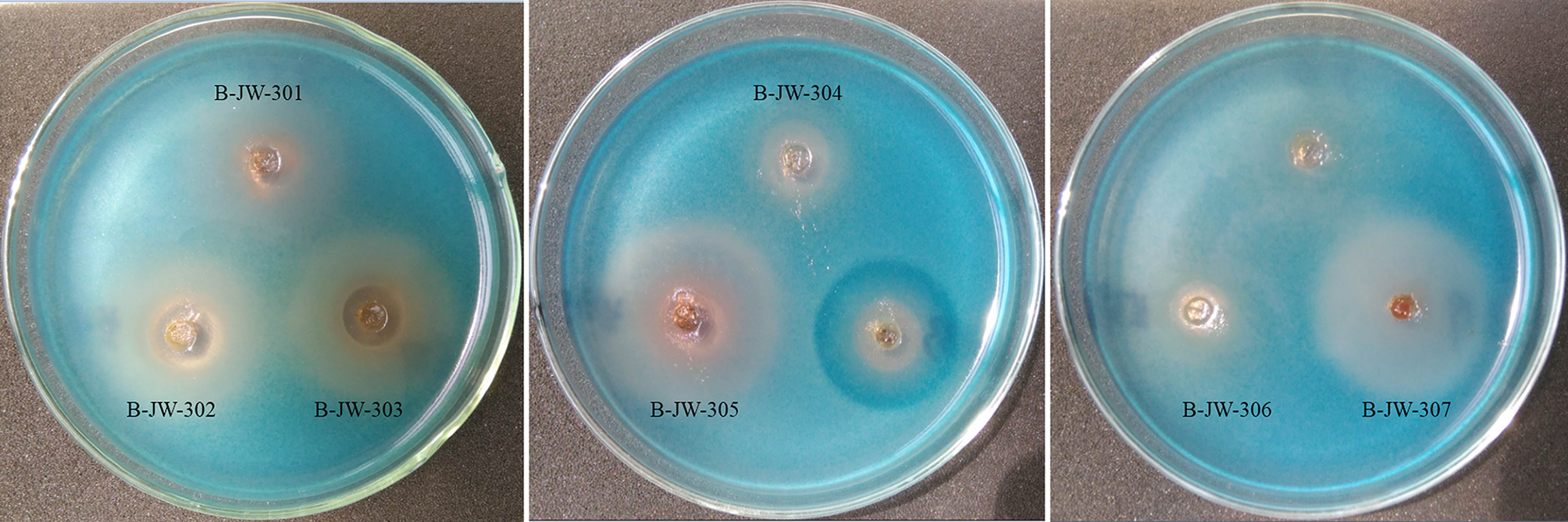

Supplement: Figure S1 — Anti-QS activities of 7 kinds of fungi using A. tumefaciens KYC55 as a reporter strain. A white halo around the well against a blue background was an indication of anti-QS zone. Overnight cultures of KYC55 (1 mL, OD620 ≈ 0.7) were aliquoted into 100 mL of warm molten AT agar medium [1.05% K2HPO4, 0.45% KH2PO4, 0.02% MgSO4·7H2O, 0.0005% FeSO4·7H2O, 0.001% CaCl2, 0.0002% MnCl2, 0.2% (NH4)2SO4, 0.2% D-mannitol, 0.8% agar, pH 7.2, autoclaved at 115°C for 15 min] and supplemented with 200 μL X-gal (50 μg mL−1, Sigma-Aldrich, USA) and 5 μM N-(3-oxooctanoyl)-L-homoserine lactone (Sigma-Aldrich, USA) and then gently mixed. The mixture was poured immediately over the surface of a solidified AT agar plate as an overlay. Wells of 5 mm in diameter were made on each plate. Finally, the fungal extract (10 μL) was added to the wells and the plates were incubated at 30°C for 24 h. [file Image1.TIF]

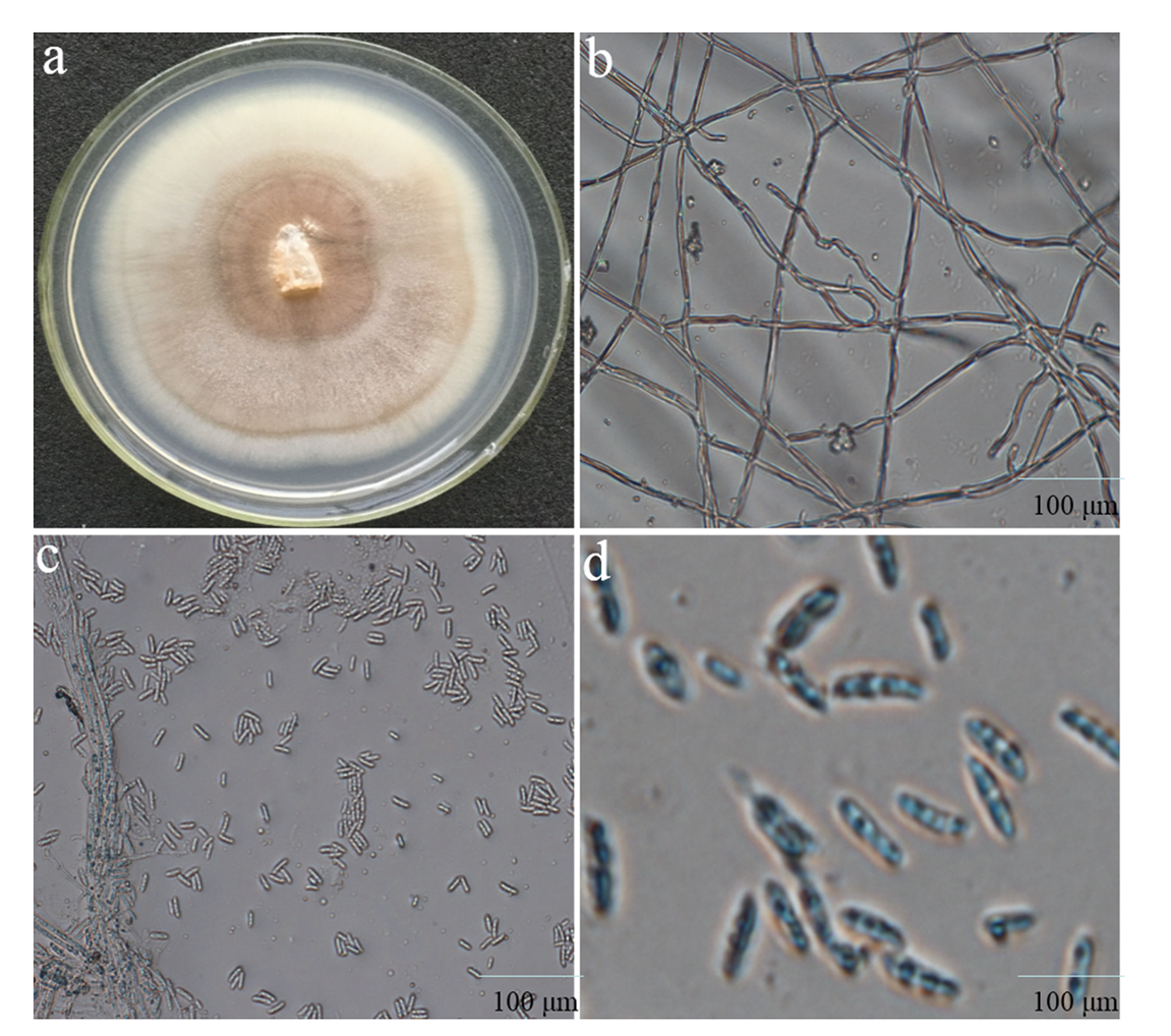

Supplement: Figure S2 — Colony morphology of P. cucumerina B-JW-304. (a) Colony on PDA after 14 d at 24°C. (b) Hyphal coil. (c,d) Septate conidia. Scale bars = 100 μm. [file Image2.TIF]

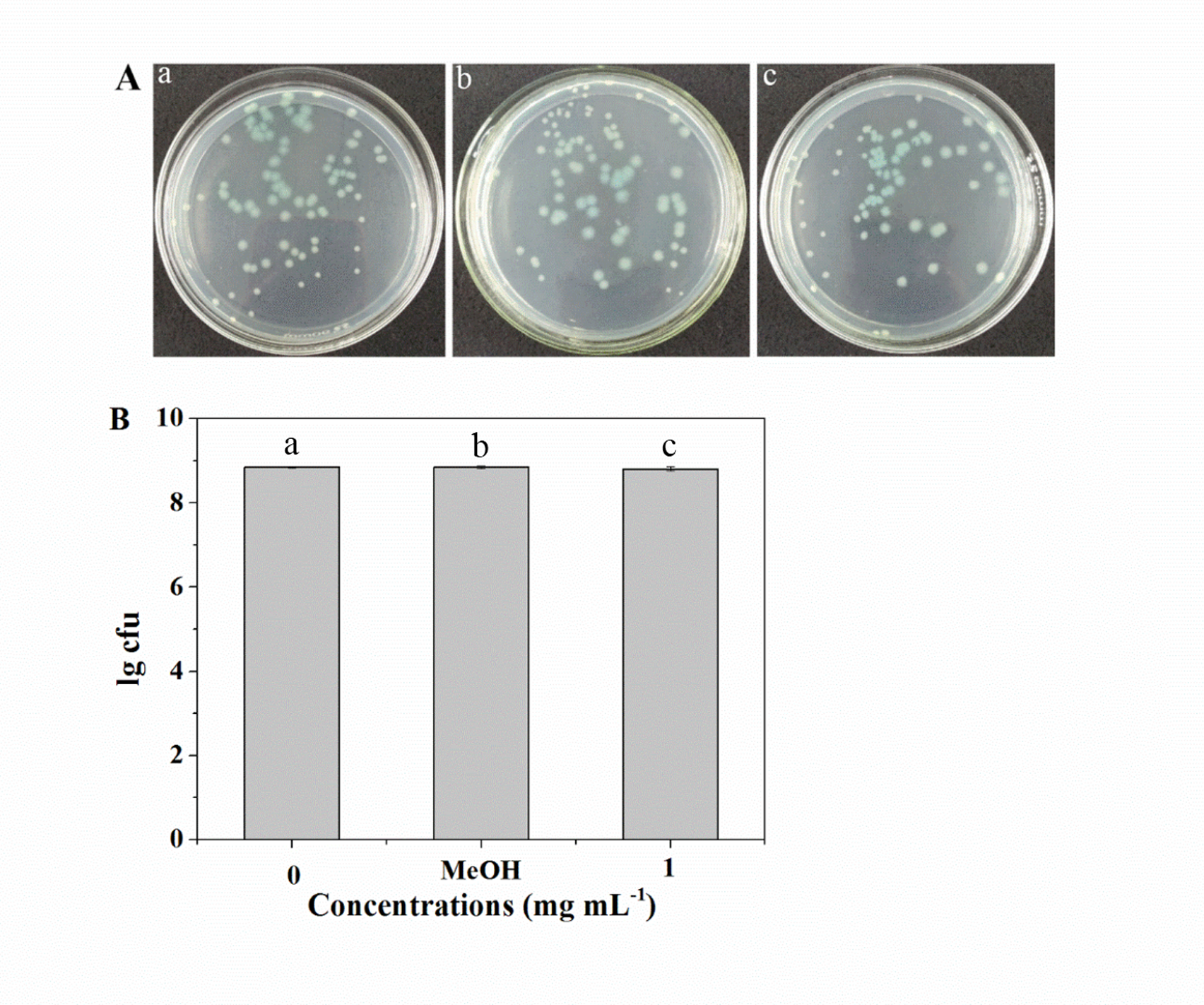

Supplement: Figure S3 — Effects of P. cucumerina extract (1 mg mL−1) on P. aeruginosa PAO1 growth. (A) Bacterial colonies of P. aeruginosa PAO1 (107fold dilution) after incubation for 24 h. (a) Distilled water control; (b) MeOH control; (c) P. cucumerina extract (1 mg mL−1) treatment. (B) Bacterial colonies number of P. aeruginosa PAO1 after incubation for 24 h with (0) distilled water, MeOH and 1 mg mL−1 of extract treatment. Error bars indicated the standard deviations of three measurements. [file Image3.tif]

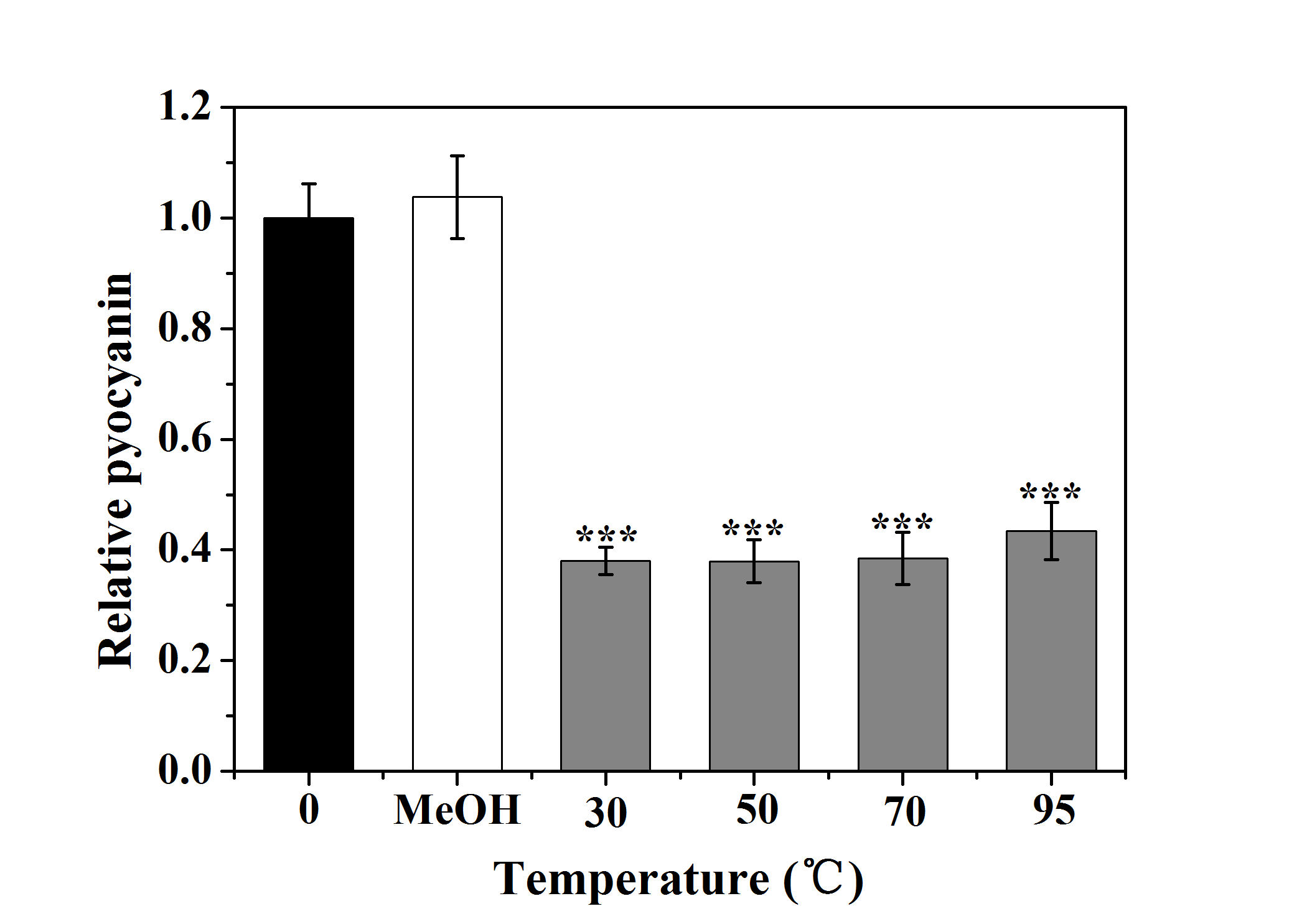

Supplement: Figure S4 — Activity analysis of P. cucumerina extract that was pretreated with heat (30–95°C) by using pyocyanin as evaluation index. Error bars indicated the standard deviations of three measurements. ***p < 0.001 vs. the control (0). [file Image4.TIF]

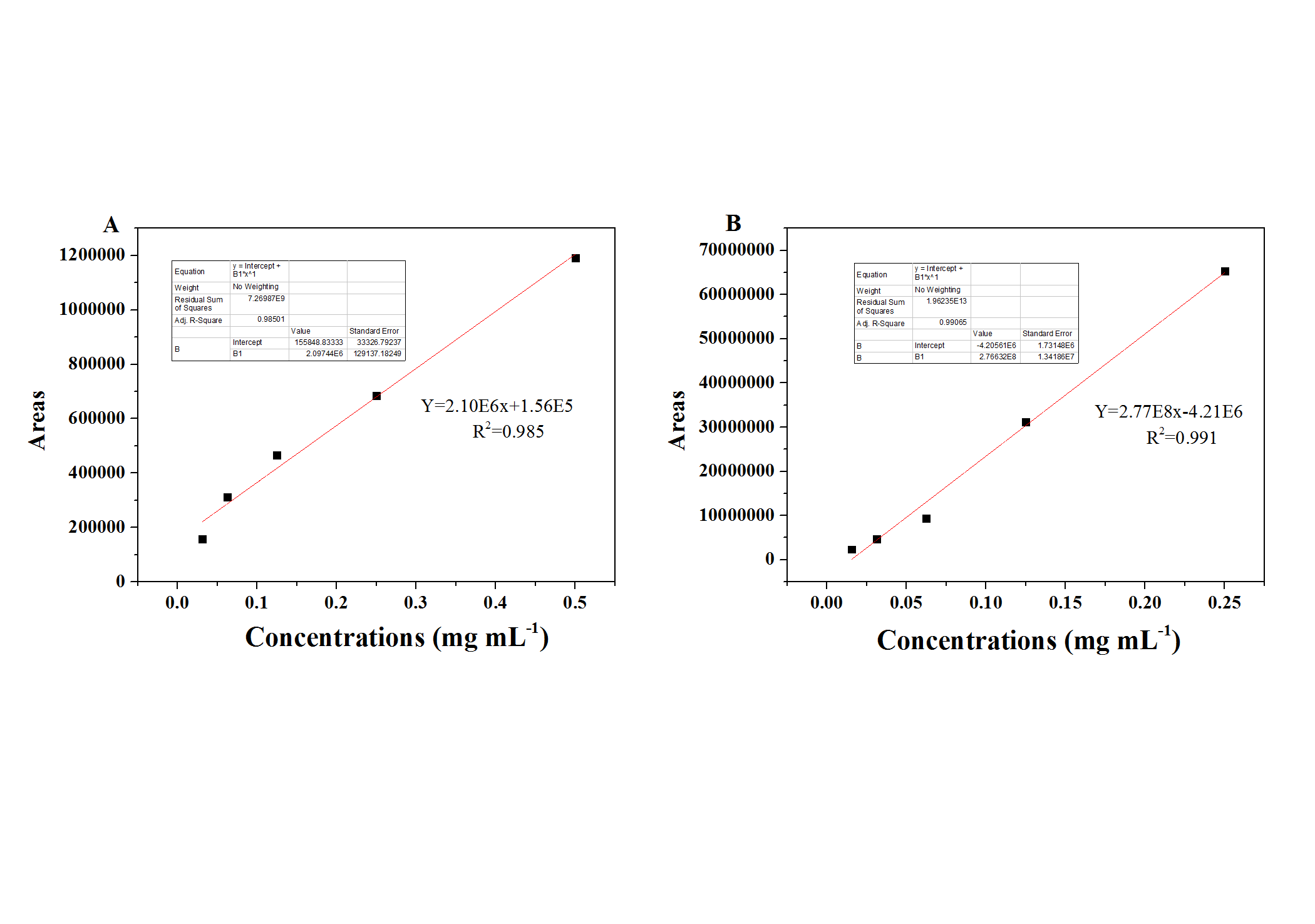

Supplement: Figure S5 — Standard curves targeting patulin (A) and emodin (B) for quantification. [file Image5.TIF]

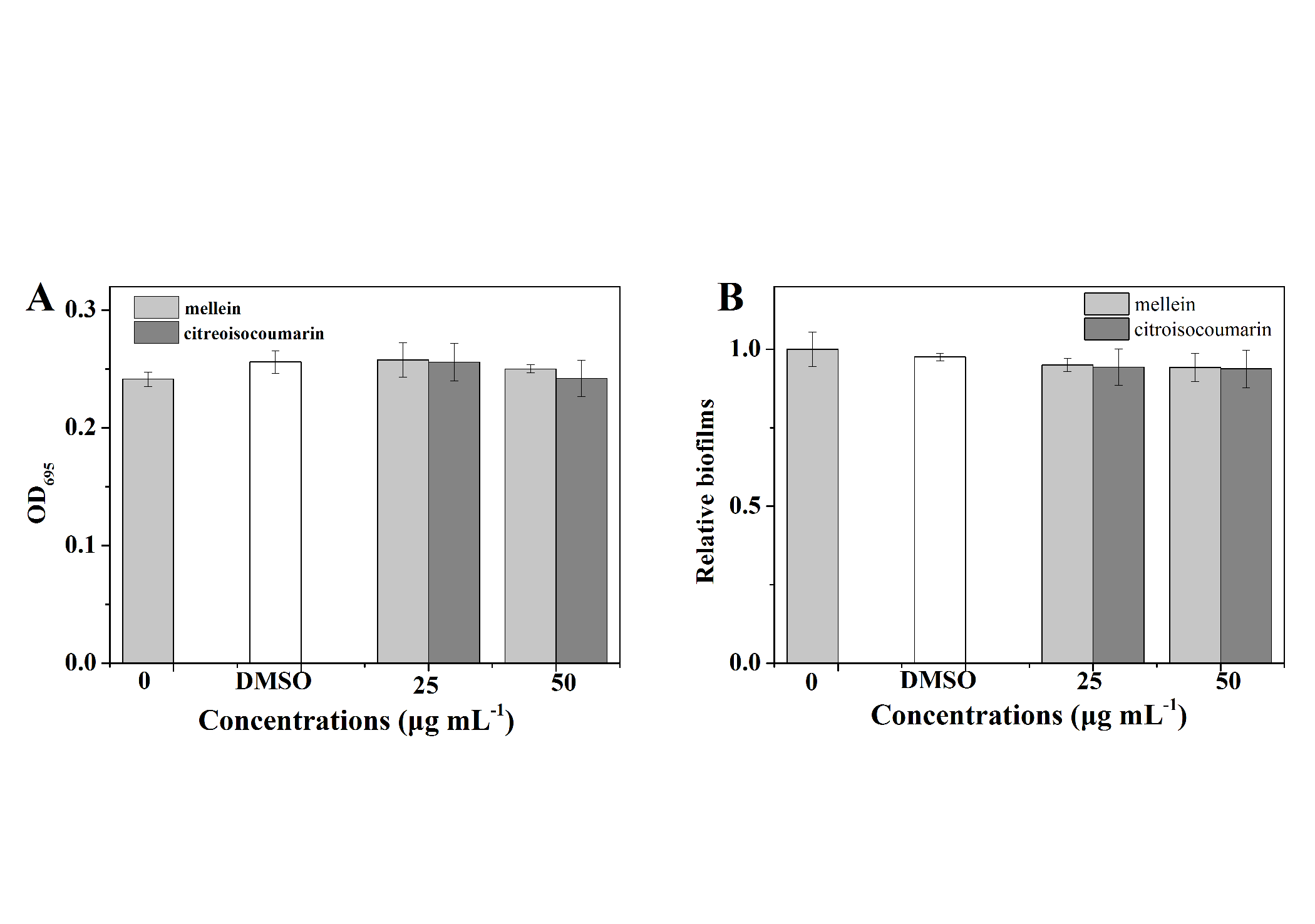

Supplement: Figure S6 — Anti-pyocyanin (A) and anti-biofilm (B) activities of mullein and citreoisocoumarin. Error bars indicated the standard deviations of three measurements. [file Image6.TIF]

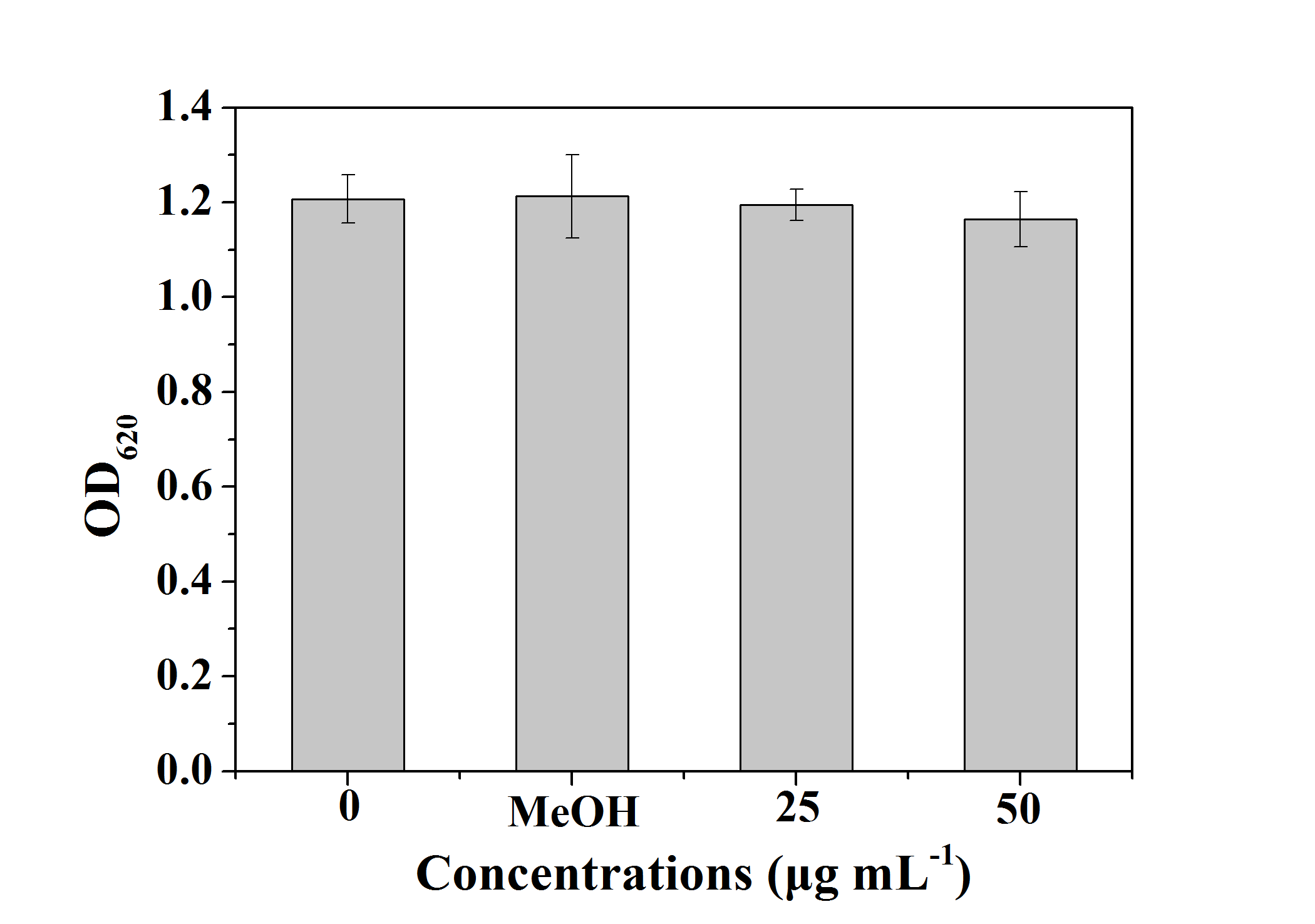

Supplement: Figure S7 — Effect of emodin on P. aeruginosa PAO1 growth. Growth at concentrations of emodin (25 and 50 μg mL−1) for 17 h in tube. Distilled water (0) and MeOH served as negative controls. Error bars indicated the standard deviations of three measurements. [file Image7.TIF]
