# Supplementary material for: Anti-Biofilm and Antivirulence Activities of Metabolites from Plectosphaerella cucumerina against Pseudomonas aeruginosa
Source: Front Microbiol. 2017 May 3;8:769. doi: 10.3389/fmicb.2017.00769 (PMC5413567; doi:10.3389/fmicb.2017.00769)
Supplement: Supplementary file 10 [file Presentation1.PDF]

Dear Prof. Yuji Morita and other editors,

On behalf of all my colleagues, I would like to thank you for giving us an opportunity to revise our manuscript. We found that the comments are constructive and helpful, so we have addressed the raised questions and revised our manuscript carefully. I sincerely wish the revised manuscript entitled **"Anti-biofilm and antivirulence activities of metabolites from *Plectosphaerella cucumerina* against *Pseudomonas aeruginosa*"** to be considered for publication in ***"Frontiers in Microbiology"***. We look forward to receiving comments from you and other reviewers. Below are the point-by-point answers (in blue colors).

Dear Dr Jia,

Your manuscript is close to be provisionally accepted. I have a lot of comments to brush up your manuscript. Please responds all of them and upload your revised manuscript onto the web.

1: Line 42: "from *P. cucumerina*" should be "*P. aeruginosa* PAO1"
Response: Thank you for your kind suggestion. The sentence "from *P. cucumerina*" corrected as "from *P. cucumerina* against *P. aeruginosa* PAO1".

2: Line 74: This sentence requires reference(s).
Response: Thank you for your kind suggestion. The reference has been supplemented in Line 74.

3: Line 90-97: Each sentence requires reference(s).
Response: Thank you for your kind suggestion. The references have been supplemented in Line 91-98.

4: Line 102: Please type "lasI" in Italics.
Response: Thanks. Corrected as suggested (Line 104).

5: Line 109-111: Each sentence requires reference(s).
Response: Thank you for your kind suggestion. The references have been supplemented in Line 112,113.

6: Line 121-122: This sentence requires reference(s).
Response: Thanks. The reference has been supplemented in Line 124,125.

7: Line 130-131: This sentence requires reference(s).
Response: Thanks. The reference has been supplemented in Line 134.

8: Line 131-132: This sentence requires reference(s).
Response: Thanks. The reference has been supplemented in Line 135.

9: Line 146: “sonicated” should be explained in more detail.
Response: Thanks. The details have been supplemented in Line 150.

10: At the section of Materials and Methods, several hand-made agars/broths were written. Please put reference(s) for each of them.
Response: Thanks. The references have been supplemented in the revised manuscript which were underlined in blue color (Line 153,158,182,190,247,261).

11: Line 154-157: “Fungal internal transcribed spacer (ITS) region (…)” requires reference(s). “Primers ITS-1 and ITS-4” requires reference(s) or description on how to be designed.
Response: Thanks. The references have been supplemented in the revised manuscript which were underlined in blue color (Line 159-161).

12: Line 187: “centrifugation” should be explained in more detail.
Response: Thanks. This sentence has been corrected as “After incubation, the fungal biomass and the fermentation broth were separated by centrifugation at 10, 000 rpm for 15 min.” (Line 193)

13: Line 188: “lyophilized” should be explained in more detail.
Response: Thanks. The details have been supplemented in Line 193-195.

14: Line 212 and others: “backdiluted” is simply “diluted”?
Response: Yes. You are right. The word “backdiluted” has been corrected as “diluted” in Line 219, 245, 276.

15: Line 237: “on tube method” should be “on the tube method”
Response: Thank you for your kind suggestion. Corrected as suggested (Line 244).

16: Line 247: What “microplate reader” did you use? Please describe it concretely.
Response: Thank you for your kind suggestion. The details have been supplemented in Line 254, 255.

17: Line 276-277: What “polystylene plate with circular glass slides” did you use ? Please describe it concretely.

Response: Thank you for your kind suggestion. The details have been supplemented in Line 285-287.

18: Line 295: It is preferable to explain what azocasein is and why it was used to make readers easier to understand your article.
Response: Thank you for your kind suggestion. The explanation have been supplemented in Line 304-309.

19: Line 305: “centrifugation” should be explained in more detail.
Response: Thank you for your kind suggestion. The details have been supplemented in Line 320.

20.: Line 316: “evaporated” should be explained in more detail.
Response: Thank you for your kind suggestion. The details have been supplemented in Line 331,332.

21: Line 378-380: This sentence is unclear. I do not understand why you selected B-JW-304. Please clarify it.
Response: Thank you for your kind suggestion. Among 41 isolated fungi, 7 kinds of fungi showed anti-QS capacities against *A. tumefaciens* KYC55 (Figure S1). However, only one fungus coded “B-JW-304” was selected for further study due to its potent antivirulence and anti-biofilm capacities against *P. aeruginosa* PAO1. No antivirulence and anti-biofilm capacities were observed in the other 6 fungi. **SO, we selected “B-JW-304” for further study.**

22: Line 385: It is preferable to explain *Plectpsphaerell cucumerina*.
Response: Thank you for your kind suggestion. The explanation has been supplemented in Line 403-409.

23: Line 390: “The MIC of extract” should be “The MIC of extract against P. aeruginosa PAO1”
Response: Thank you for your kind suggestion. Corrected as suggested (Line 413).

24: Line 406-410: These sentences seem to be repetition of Introduction. Please revise them to be shorter.
Response: Thanks. These sentences have been reorganized in Line 429.

25: Line 432: “the formation of biofilm by *P. aeruginosa* is correlated with EPS” requires reference(s).
Response: Thanks. The reference has been supplemented in Line 451, 452.

26: Line 450: “a standard quantitative biofilm assay method” requires reference(s).
Response: Thanks. The reference has been supplemented in Line 470.

27. Line 464 & 465: “virulence factors” should be “virulence factors of P. aeruginosa”
Response: Thanks. Corrected as you suggested (Line 484-486).

28. Line 469: This sentence requires reference(s).
Response: Thanks. The reference has been supplemented in Line 490.

29. Line 474: This sentence requires reference(s). Is there need to add “in P. aeruginosa” to this sentence?
Response: Thanks. The reference has been supplemented and “in *P. aeruginosa*” has been added in Line 494, 495.

30. Line 488-489: This sentence requires reference(s).
Response: Thanks. The reference has been supplemented in Line 510.

31. Line 583: This sentence requires reference(s).
Response: Thanks. The reference has been supplemented in Line 604, 605.

32. Line 599-601: This sentence requires reference(s).
Response: Thanks. The reference has been supplemented in Line 622.

33. Line 620: This sentence requires reference(s).
Response: Thanks. The reference has been supplemented in Line 642.

34. Line 676: “reporter strain” should be “a reporter strain”?
Response: Thanks. Yes, you are right. Corrected as you suggested (Line 698,699).

35. Fig. 1: Italic Words should be written in Italic.
Response: Thank you for your valuable suggestion. Corrected as suggested.

36. These articles bellow might be appropriate as references of this manuscript. Please read through them and cite some of them.

1: Liu YC, Chan KG, Chang CY. Modulation of Host Biology by Pseudomonas aeruginosa Quorum Sensing Signal Molecules: Messengers or Traitors. Front Microbiol. 2015 Nov 9;6:1226. doi: 10.3389/fmicb.2015.01226.
Response: This reference has been supplemented in Line 96,97.

2: Chan KG, Liu YC, Chang CY. Inhibiting N-acyl-homoserine lactone synthesis and quenching Pseudomonas quinolone quorum sensing to attenuate virulence. Front Microbiol. 2015 Oct 19;6:1173. doi: 10.3389/fmicb.2015.01173.
Response: This reference has been supplemented in Line 96,622.

4: Michalska M, Wolf P. Pseudomonas Exotoxin A: optimized by evolution for effective killing. Front Microbiol. 2015 Sep 15;6:963. doi:10.3389/fmicb.2015.00963.

Response: This reference has been supplemented in Line 606.

5: Morita Y, Tomida J, Kawamura Y. Responses of Pseudomonas aeruginosa to antimicrobials. Front Microbiol. 2014 Jan 8;4:422. doi: 10.3389/fmicb.2013.00422.
Response: This reference has been supplemented in Line 76.

6: Imperi F, Leoni L, Visca P. Antivirulence activity of azithromycin in Pseudomonas aeruginosa. Front Microbiol. 2014 Apr 22;5:178. doi: 10.3389/fmicb.2014.00178.
Response: This reference has been supplemented in Line 78,79.

7: Please click bellow which lists articles which might be references.
https://www.ncbi.nlm.nih.gov/pubmed/?term=(%22Frontiers+in+microbiology%22%5BJournal%5D)+AND+quorum+sensing++AND+Pseudomonas+aeruginosa
Response: Some of the references have been supplemented in the manuscript.
